# Biological and synthetic mesh use in breast reconstructive surgery: a literature review 

**DOI:** 10.1186/s12957-016-0874-9

**Published:** 2016-04-21

**Authors:** Hugh Logan Ellis, Oluwatosin Asaolu, Vivien Nebo, Abdul Kasem

**Affiliations:** Medway Maritime Hospital , Gillingham, Kent UK; King’s College London University, London, UK

**Keywords:** Breast reconstruction, Synthetic, Biological, Matrix, Mesh, Aesthetic outcomes, Cost, Complications

## Abstract

Mesh use in surgical breast reconstruction is becoming increasingly common; however, there is still no consensus on whether synthetic matrices or biological matrices produce the best outcomes. This review analyses these outcomes, namely the differences in aesthetic outcomes, cost, and the rates of the most commonly reported complications.

The results indicate that breast reconstruction with a synthetic matrix produces comparable aesthetic outcomes to a biological matrix, with lower costs and complication rates. The individual results for complication rates show that biological matrixes are associated with lower infection rates and slightly lower capsular contracture, but higher haematoma rates, and slightly higher rates of skin necrosis and explantation—although many had post-op radiotherapy.

The majority of the studies evaluated used biological matrices, and there are no randomised controlled trials directly comparing the two types of meshes; definite conclusions cannot be drawn from the available evidence. The authors suggest that a randomised controlled trial comparing these outcomes in synthetic and biological matrix use is needed.

**Electronic supplementary material:**

The online version of this article (doi:10.1186/s12957-016-0874-9) contains supplementary material, which is available to authorized users.

## Background

In England, around 40 % of breast cancer patients opt for mastectomy as their primary therapeutic management [1]. Of the 18,000 mastectomies performed in England and Wales [2], 21 % undergo immediate reconstruction [1].

Surgical breast reconstruction (BR) post-mastectomy was first performed in 1895 by Vincent Czerny using a “fist-sized lipoma from the patient’s flank” [3]. Since then, various improvements have been made to BR, including the introduction of immediate—as opposed to delayed—reconstruction, which has shown significantly improved aesthetic outcomes [4], psychological health, and increased quality of life [5]. Numerous surgical techniques have been developed, including the use of autologous tissue flaps, most commonly from the abdomen and the back or silicone implants with or without prior insertion of a tissue expander (TE) [6]. As with any surgical procedure, there are complications. These will be discussed in some depth in this review.

More recently, biological and synthetic matrices have emerged as a useful adjunct to BR.

A biological mesh—also referred to as an acellular dermal matrix (ADM)—is a scaffold of dermis produced from cadaveric human (Alloderm®, Allomax®, FlexHD®, DermaCell®), porcine (Strattice®, Permacol™), bovine (SurgiMend®), or bovine pericardium (Veritas®) tissue that is stripped of its antigenic cells through specialised processing [7]. The biological scaffold allows rapid host revascularisation and cell repopulation arguably facilitating a good surgical outcome.

**Fig. 1:**
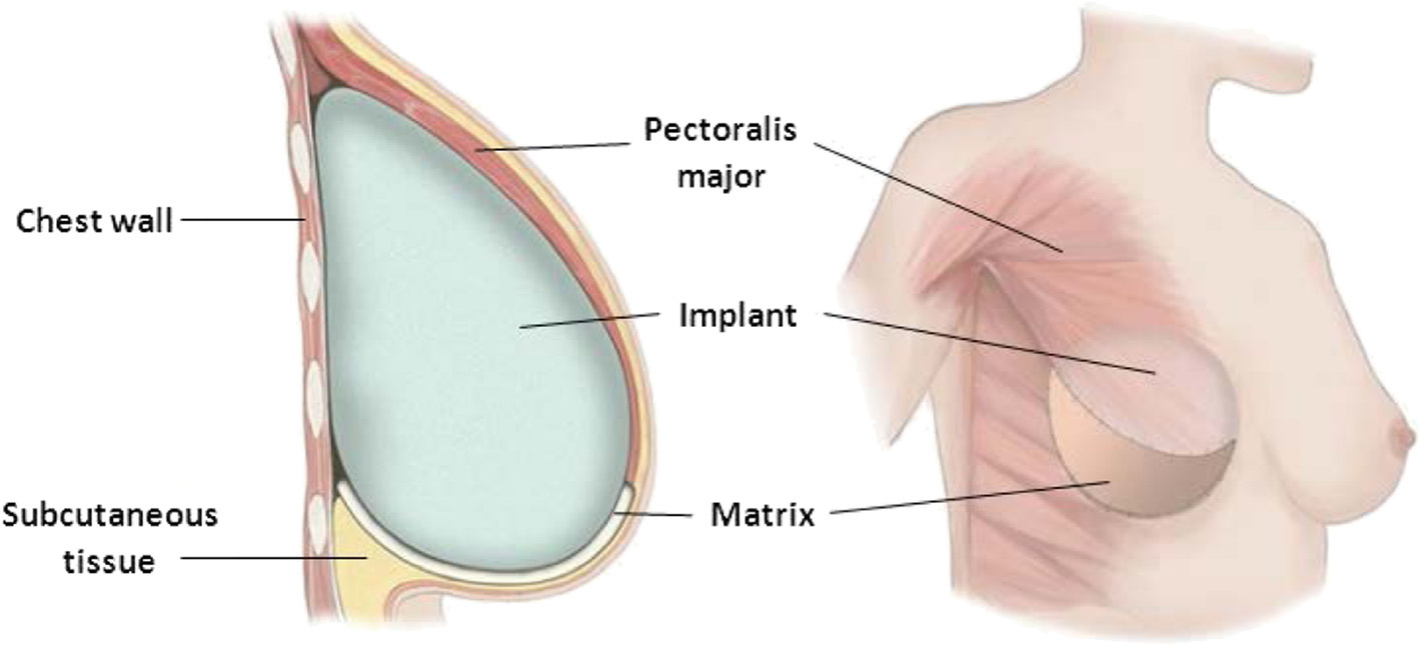
Placement of biological matrix. Adapted image showing the placement of the matrix (biological or synthetic) between the inferior pole and the aponeurosis of the pectoralis major muscle. The matrix is supporting the lower pole of the breast, while expanding the implant pocket, providing increased coverage of the implant [97]

Most studies have reviewed biological matrices in implant-based reconstruction, acting as an extension of the pectoralis major muscle [8, 9]. By attaching to the inferior-lateral pole of the muscle, the mesh expands the space available for the insertion of an implant, filling the void left between the muscle and fascia, thereby creating a natural inframammory fold (see Fig. 1) [10]. This technique provides additional cover and support inferiorly, enabling faster tissue expansion, larger implant volumes, and improvement of lower pole projection [11].

Despite the advantages of ADMs, they do not come without complication. These include infection, cellulitis, seroma, haematoma, skin flap necrosis, wound dehiscence, capsular contracture, implant extrusion/exposure, and explantation/implant loss [12]. In addition, studies have shown adverse effects associated with radiotherapy, as well as high cost [8, 9] when ADMs are used.

Recent studies are now investigating the use of low-cost synthetic matrices in BR as an alternative to ADMs [10]. Synthetic matrices are made from plastic-like material: absorbable (Vicryl), long-term absorbable (TIGR®), or non-absorbable (titanium-coated polypropylene mesh (TiLOOP®)). Although these synthetic meshes play a similar role to ADMs in BR, it remains unclear whether the complication rates between synthetic and biological matrices differ, as currently, there are no studies comparing them.

This literature review will compare synthetic and biological meshes use in BR, comparing differences in aesthetic outcomes, cost, and the most commonly reported complications.

## Method

A literature search was conducted with the view of identifying articles relevant to a retrospective review of biological and synthetic matrices used in BR post-mastectomy. PubMed was used as a search engine. The terms entered were the following: “breast reconstruction AND mesh,” “breast reconstruction AND acellular dermal matrix,” “breast reconstruction AND Strattice,” “breast reconstruction AND synthetic mesh,” “breast reconstruction AND Vicryl,” “breast reconstruction AND TIGR,” and “breast reconstruction AND TiLOOP.” The results from this search were limited to articles published between January 2004 and March 2014, giving 519 results.

These articles were then screened for eligibility according to the inclusion and exclusion criteria. Studies involving BR post-mastectomy (whether immediate or delayed) that used a mesh for the reconstruction were included. In addition, some in vitro and in vivo articles, as well as those providing histological analysis, were included. Papers were excluded if their focus was the use of mesh on the following areas: abdominal reinforcement post-BR, chest wall reconstruction, and the revision of primary and secondary deformities.

The study method, number of subjects, type of mastectomy (skin-sparing, nipple-sparing, total, modified radical, radical, or extended radical), type of mesh (biological or synthetic), type of reconstruction (immediate or delayed), surgical technique (direct-to-implant/single-stage or TE-based/two-stage), and the results including any complications were all recorded.

A full table of all the literature evaluated in this review can be found as Additional file 1.

## Results

Each study was evaluated for specific outcomes, namely aesthetic outcomes, cost, complication rates and effects of radiotherapy.

### Aesthetic outcomes

The literature shows positive aesthetic outcomes associated with the use of ADMs in surgical BR [11, 13–19]. Specifically, Vardanian et al. [13] reported higher overall aesthetic outcomes when an ADM was placed, compared to no matrix, with statistically reduced bottoming-out (*p* = 0.002), rippling (*p* = 0.011), and mechanical shift (*p* = 0.011) [13]. A retrospective review by Gamboa-Bobadilla et al. [14] reported good and excellent aesthetic outcomes in 91 % of subjects just 14 months after an implant-based BR with ADM. In line with this, Spear et al. [15] reported aesthetic outcomes comparable to non-surgery controls after undergoing expander-based BR. While Forsberg et al. [11] found statistically significant improvements in natural contour, symmetry of shape and size, position on chest wall, and overall aesthetic outcomes, when ADM was used in expander-based BR compared to without.

Similar aesthetic results have been reported with the use of synthetic matrices. A study by Kim and Cho [20] evaluating the use of the absorbable Vicryl mesh in implant-based BR described excellent/good cosmetic outcome in 91 % of patients. While a retrospective review by Rietjens et al. [21] reported an average symmetry of 7.56, a patient satisfaction of 7.75, and a surgeon cosmetic evaluation of 7.60 (all rated out of 10), 28 months after expander-based BR using a non-absorbable mesh (Mersilene).

### Cost

The high cost of matrices is one factor that continues to be a deterrent to their use in BR [22]. A single sheet can range anywhere between $1825 [9] and $4856 [8], depending on its size and thickness, although increasing competition between types of ADM has helped reduced this [22]. An advantage of ADM use is direct-to-implant BR. This technique does not require tissue expansion before implant insertion, prevents the donor site morbidity and lengthy recovery time associated with autologous flap reconstruction, and reduces operating time substantially, compared with autologous flap and expander-based BR [23]. Johnson et al. [23] showed that the placement of a unilateral implant with Strattice mesh produced a total cost of £3685 for the surgery per patient, which was significantly lower than unilateral TE (£4985) and latissimus dorsi flap (£6321) implant-based BR surgery. These numbers indicate that despite the extra cost of an ADM, it may reduce the overall cost of surgery.

Newer studies have begun to consider the use of synthetic matrices as a low-cost alternative to ADMs in BR. A recent retrospective review conducted by Tessler et al. [10] revealed direct material cost savings of $172,112 over a 10-month period with the use of synthetic mesh (Vircyl) in implant-based BR, when compared directly to ADM expenses at the authors’ institution.

### Complication rates

Mesh use in BR is associated with many complications [24–29]. Of these, infection, seroma, haematoma, capsular contracture, skin flap necrosis, and explantation or implant loss were the most commonly reported.

It has been shown several times that ADM use is associated with an increased risk of complications in BR surgery and more recent studies have analysed the complication rate when synthetic meshes are used [10, 20, 21, 24–26, 30, 31]. However, there are currently no articles in the literature directly comparing the complication rates between these two types of mesh.

Patient characteristics have been associated with increased complication rates in BR in general, with or without mesh use [32–34]. These include age (>65 years), large breasts (>600 g), obesity (body mass index >30), smoking, diabetes, hypertension, and long drain removal time. Although it is important to consider these factors, it is clear that these occur regardless of whether or not a mesh is used. Appropriate patient selection and individualisation of reconstructive options are needed to account for any co-morbidities and risk factors that may be present among patients.

### Infection

Infection is one of the most common complications seen in both biological and synthetic mesh use, which often leads to tissue necrosis, and may result in explantation, revision, or even complete loss of implant [10, 24, 35, 36]. Dieterich et al. [24] have conducted the largest study (*n* = 207, 231 cases) using a synthetic mesh (TiLOOP®) in implant-based BR to date, producing an overall infection rate of 6.1 %, of which only 1.7 % needed revision. Other studies of synthetic mesh use in BR have revealed lower rates of infection (1.3–4.7 %) [10, 20, 21, 26, 31], and two papers reported no cases of infection at all [25, 31].

A large retrospective review by Ibrahim et al. [37] of 19,100 subjects undergoing implant-based BR showed a lower infection rate of 3.3 % in subjects where ADMs were used (*n* = 3301). A meta-analysis by Kim et al. [38] of 19 papers (*n* = 2037) comparing the submuscular TE technique with human ADM BR showed an infection rate of 5.3 % with ADM use. This was reported as a relative risk of 2.47 (95 % confidence interval (CI), 1.71–3.57), when compared to the submuscular group. The remaining studies analysed reported a rate ranging between 0.2 and 35.8 % [11, 13, 23, 27, 39–59], with four studies reporting no complications of infection [16, 60–62]—although this could be attributed to the relatively low subject numbers.

An explanation for these findings is offered by a high-throughput assay comparing synthetic (Prolene and Vicryl) and biological (AlloDerm and FlexHD) matrices as substrates for bacterial adhesion, which concluded that *Staphylococcus aureus* adhered more readily to ADMs than synthetic matrices [63]. In addition, post-operative antibiotic prophylaxis following mesh BR, as trialled by Avashia et al. [64], has shown some promise in reducing infection rates from 31.6 to 11.1 % (*p* = 0.004) in participants who underwent implant-based reconstruction using ADM.

### Seroma and haematoma

Seroma and haematoma are commonly occurring complications associated with surgical BR and can both lead to an increased risk of infection and tissue necrosis, particularly when large enough to require drain insertion [28] which carries the risk of TE or implant puncture.

**Fig. 2:**
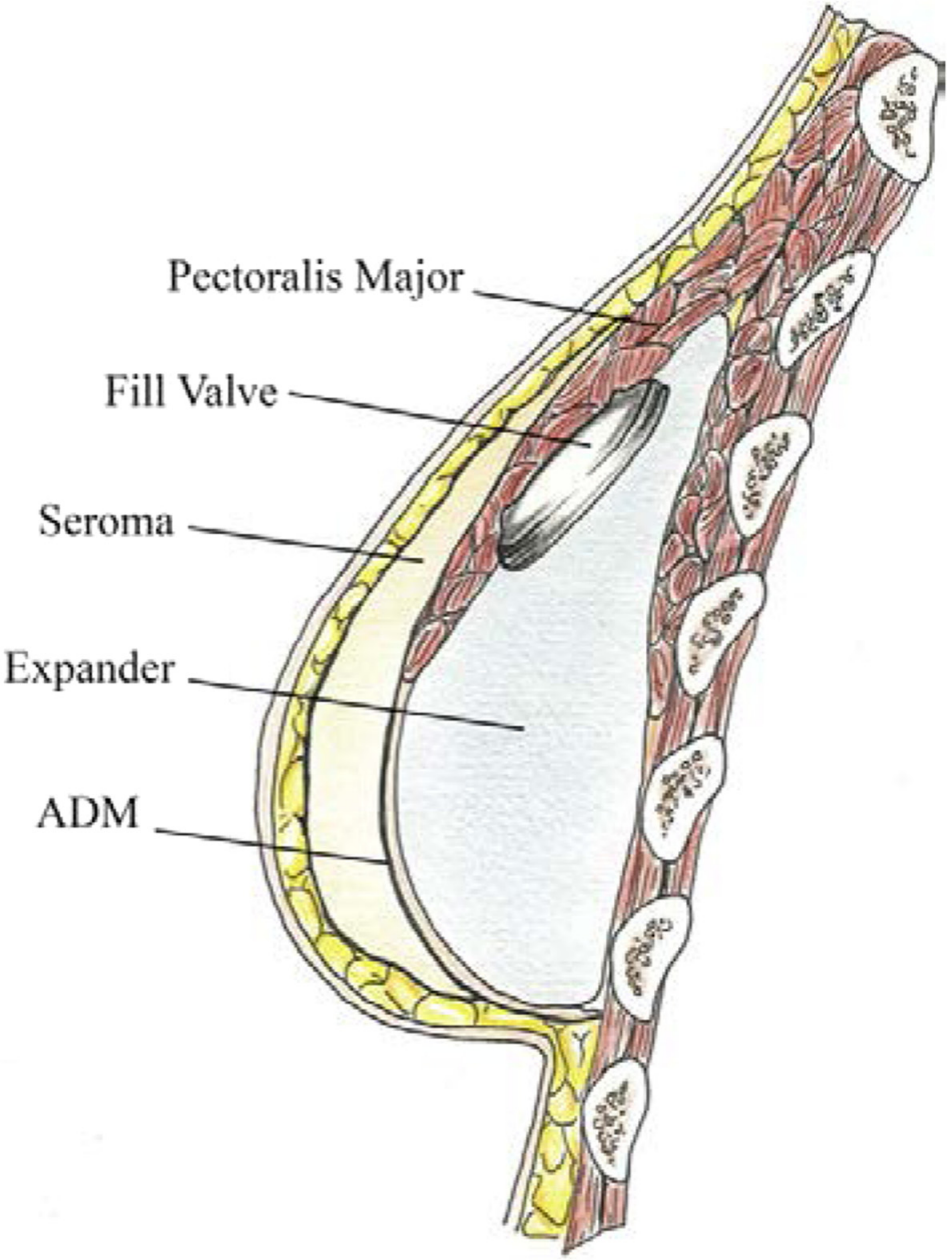
Seroma formation. Animated image of seroma development between breast tissue envelope and pectoralis major/mesh (ADM) layer [28]

Seroma is an especially detrimental problem in TE-based BR, where its development between the pectoralis major muscle/mesh layer and the breast tissue envelope (see Fig. 2) may result in a poor aesthetic outcome upon exchange to implant [28].

Haematoma formation is thought to occur in the immediate post-operative period as a result of trauma during surgery—although the use of electrocautery has significantly reduced this [65]—or as a late complication due to small tears in the capsule formed around the TE/implant, often after physical trauma [66–68].

Seroma formation in association with synthetic mesh use in surgical BR has been evaluated in six different studies [10, 20, 24–26, 30] and ranges from no seroma development [10] to 5.7 % [20]. Dieterich et al. [24], the largest (*n* = 207, 231 cases) and therefore perhaps the most reliable study of synthetic mesh (TiLOOP) use to date, revealed an overall seroma rate of 4.8 %, although only 1.4 % required revision. Dietrich et al. also found the highest rate of haematoma, at 9.5 %, treated with either puncture or compression. Other studies looking at the use of synthetic mesh in BR showed haematoma rates of 0–4.7 % [10, 25, 26].

The presence of seroma as a complication of ADM use in BR was found to range widely between 1.5 % [54] and 24.3 % [23], in the studies that evaluated it as a complication. The meta-analysis by Kim et al. [38] was the largest (*n* = 2037) study to report the rates of seromas in BR with ADM. Their results showed a seroma rate of 4.8 % with a relative risk of 2.73 (95 % CI, 1.67–4.46). Haematoma formation after BR with ADM produced a smaller range of 0–11.1 % [17, 27, 29, 69, 70], the highest of which was reported by Moyer et al. [27], where all participants received radiotherapy.

### Capsular contracture

Capsular contracture is described as the formation of a fibrous capsule around the implant, which may contract, compressing the implant as it thickens progressively, resulting in a hard breast with deformed contouring of the surrounding skin [71]. This may result in severe pain, due to nerve entrapment, or muscle mobility interference. Although the aetiology is unclear, some believe that it is initiated by blood products like haematomas [72], while others consider infection and chronic inflammation to be most likely cause [73]—either by bacteria colonisation from within the ductal breast tissue or the skin.

The Baker classification system is used to assess the severity of capsular contracture in a clinical setting, with classes ranging from I to IV [74]. A clinically significant outcome is usually only associated with a Baker class of III (moderate firmness) or IV (symptomatic with severe firmness), although most studies note all levels of contracture. Before the introduction of ADM use, high rates of capsule contracture were frequently reported [72]. With the advent of ADM, studies have reported significantly lower rates of capsular contracture [13, 15, 18, 19, 32, 39, 49, 51, 69, 75], although no studies have directly compared the incidence of capsular contracture between synthetic and biological matrices.

Of the five studies that evaluated the rate of capsular contracture in cases of BR with synthetic mesh, Tessler et al. [10] reported the lowest rate of contracture needing revision, at 1.3 %, while the highest rate of severe contracture leading to pain was 8.6 %—seen by Kim and Cho [20]. Rietjens et al. [21] reported the highest overall rate of capsule contracture at 68.4 %; however, only 13.7 % of these were graded as Baker class III or IV. It should also be noted that a non-absorbable mesh (Mersilene) was used—a type of synthetic mesh that has been reported to produce rigidity [30]. Loustau et al. [25] were the only authors to report no capsular contracture.

In keeping with the literature described above, the majority of the studies reported little or no capsular contracture (six studies), ranging between 0.4 and 8.1 % [11, 39] when ADM was used. Moyer et al. [27] studied the effect of radiation on ADM and capsule formation, revealing a very high rate of Baker class III/IV capsular contracture of 33.3 % (*n* = 27).

Studies of the histological changes ADM use [76–79] support the hypothesis that ADMs—due to their lack of antigenic epitopes—provide a barrier to the host immune response against a foreign body (the expander/implant) and reduce capsular formation and contracture risk [76]. Komorowska-Timek et al. [79] studied the effect of an ADM on capsule formation with and without radiation and concluded that ADM reduces radiation-related inflammation and pseudoepithelium formation, resulting in slower progression of capsular formation and contraction. The incidence of capsular contracture in human subjects where ADM has been used also shows significantly reduced contracture problems, histologically [77] and clinically [78].

Few studies have evaluated the histological outcomes upon use of synthetic matrices in surgical BR. Dieterich et al. [80] produced a case report and histological analysis on the use of TiLOOP® in BR, showing mild inflammation, indicating a low risk of capsular contracture.

### Skin necrosis

Skin necrosis and breakdown in the context of BR is multifactorial; patient co-morbidities, thin mastectomy skin flaps, or overexpansion may contribute [12]. When overexpansion occurs, there is a risk of loss of vascular supply to the overlying mastectomy skin flap, resulting in ischaemic changes [81].

Three studies have reported the occurrence of skin necrosis in BR with synthetic mesh. Becker and Lind [30] conducted a retrospective review using the TIGR matrix in BR, revision, and cosmetic surgery, finding skin flap necrosis in 1.8 % of participants (*n* = 62, 112 reconstructions). Two separate studies conducted by Dieterich et al. [26] and [24] showed skin necrosis of 2.4 and 4.3 % (0.4 % of which was native to the mastectomy flap), respectively.

Clemens and Kronowitz [41] showed higher rates of skin necrosis in BR with ADM (34.2 %). However, of the remaining studies collected evaluating this complication with ADM use, only five other studies produced rates in this vicinity (23.7–28.3 %) [18, 29, 36, 45, 82]. All other studies found skin necrosis rates below 12.5 % [19, 42, 44, 75, 83], with Strattice showing skin necrosis rates of only 1.4–2.9 % [44, 84].

### Explantation and implant loss

All of the above complications, if severe enough, may lead to TE or implant explantation, often resulting in complete implant loss. Dieterich et al. [24] revealed the highest (8.7 %) rate of implant loss of the four studies evaluating this in BR when synthetic mesh was used [10, 21, 24, 26], while Tessler et al. [10] reported the lowest implant loss rate of 1.3 %, when using the Vicryl mesh.

Thirty-nine studies evaluated explantation and implant loss in BR with ADM, ranging from 0 to 33.3 % [18, 27, 29, 85]. Despite this high value, majority of the studies within this range showed comparable rates of implantation loss to those with synthetic mesh use [42, 44, 76, 86].

### Effect of radiotherapy

Radiotherapy has a significantly detrimental effect on most BR outcomes, whether given pre- or post-reconstruction [15, 20, 30, 49, 58, 82] and diminishes neovascularisation and mesh incorporation into the host [87].

In regard to aesthetic outcome post-radiotherapy, Kim and Cho [20] reported a significant reduction in excellent/good aesthetic outcomes from 91 to 60 %, when the Vicryl mesh was used in implant-based reconstruction. Similarly, Hanna et al. [49] reported reduced patient satisfaction after their expander-based reconstruction with ADM.

In addition, increased rates of complications in participants who received radiotherapy before and after surgery have been reported in several studies [15, 24, 30, 82]. Becker and Lind [31] showed that 44 % of irradiated subjects that received radiotherapy developed post-reconstruction complications when TIGR was used. Dieterich et al. [24] reported that the TiLOOP mesh was more palpable in irradiated patients but barely palpable in non-irradiated patients who had very thin mastectomy flaps.

Spear et al. [15], Brooke et al. [58], and Pestana et al. [82] all showed a significant increase in overall complication rates when ADMs were used. Spears et al. found a difference in complications of 42.1 % (*p* = 0.002) between irradiated and non-irradiated patients. Other studies showed specific increases in infection [88] and implant loss [53, 89, 90] when radiation was given to subjects who had BR with an ADM [88, 89]. This correlates with results again by Spear et al. [59], showing higher rates of contracture and increased rates of implant loss upon expander exchange, in patients to whom radiotherapy was given.

Studies in animals [79] and human subjects [27, 40, 57] have reported a protective effect against radiotherapy associated with ADM in comparison to without mesh. Moyer et al. [27] showed a reduced elastosis and chronic inflammation of the breast capsules of six patients, when compared to non-irradiated controls. Similarly, Seth et al. [57] found that ADM BR was associated with lower risk of all complications due to radiation, when compared to non-ADM BR.

## Discussion

The major limitation of this review is that no studies known to the authors have directly compared the use of synthetic and biological matrices in BR. It is therefore very difficult to make any concrete comparisons between the two types of mesh. However, some conclusions have been drawn relating to the outcomes evaluated in the results, using the limited data available.

Aesthetic outcome is a commonly reported endpoint in BR and influences patient satisfaction post-BR significantly [21]. The results above showed high rates of positive aesthetic outcomes in both biological and synthetic matrices.

Although synthetic matrices are being evaluated as cheaper alternatives to biological matrices, the benefits of using ADMs in BR may outweigh the high costs discussed in the results [22]. One positive feature that distinguishes ADMs from synthetic matrices is the ability to incorporate into the host tissue [91]. This incorporation has been shown to parallel normal wound healing [92], allowing for tissue remodelling and regeneration [93].

The results comparing complication rates between meshes must be discussed separately. It appears that the use of synthetic matrices in BR is associated with a lower rate of infection compared to ADM use. However, no statistical methods have been used to take into account any outliers that may have increased the wide range of infection rate when using biological mesh. In addition, it appears that not all ADMs are equal; long-term sterility could be one of the reasons for the wide range of infection rates seen, with studies showing reduced infection rates when comparing sterile to non-sterile ADMs used in BR [61], even when aseptic techniques are strictly adhered to [36]. Notably, the number of studies found evaluating the rate of infection when using ADM is eightfold more than those reporting infection as a complication of BR with synthetic mesh. Therefore, while synthetic matrices in BR seem to have a lower associated infection rate, more studies evaluating this are needed to make a definite conclusion.

This leads to the second major limitation—the paucity of studies reporting the outcomes associated with the use of synthetic mesh in BR. Although various studies have evaluated the use of synthetic mesh in abdominal hernia repair [94–96], more research is needed into breast reconstructive surgery. It is likely that this not only affected the evaluation of infection rates but also influenced all of the outcomes to an extent—particularly capsular contracture, skin necrosis, and explantation/implant loss.

Two studies evaluating capsular contracture produced significantly outliers; the synthetic mesh evaluated by Rietjens et al. [21] may have caused bias in the clinical evaluation of capsular contracture due to its rigidity, leading to the high contracture rates reported above. The second outlier was reported by Moyer et al. [27], with a capsular contracture rate fourfold higher than the other 17 studies with ADM, which was attributed to a small sample size (*n* = 27). Upon exclusion of these outliers, a slightly higher range of capsular contracture was reported with synthetic mesh, suggesting that the biological matrix may give a small advantage against capsular contracture. However, many more studies have been published analysing the association between BR with ADM than with synthetic mesh.

Similarly, many more studies have evaluated skin necrosis and explantation/implant loss in association with biological than synthetic matrices. This must be taken in account when looking at the results of these two complications and may indicate that the use of synthetic mesh in BR produces lower rates of skin necrosis and explantation. It is again unlikely that a true representation of the rates of explantation/implant loss with ADM has been obtained, as the higher end of the range (0–33.3 %) was due to nine patients in Moyer et al. [27], all of whom were exposed to radiotherapy, which as discussed has detrimental effects on the results. It is hoped that as the evidence of the use of synthetic mesh increases, a more accurate picture of the actual rates of skin necrosis, explantation/implant loss, and capsular contracture will be possible.

Although the ranges produced from the data evaluating seroma formation significantly differ, the two largest studies evaluating this complication when using synthetic and biological matrices in BR obtained the exact same rate of this complication [24, 38]. This suggests that there may be little difference between the rates of seroma occurrence when comparing the two types of mesh. On the other hand, the rates of haematoma formation were greater in BR with ADM compared to synthetic matrices. However, it is unclear how much influence the effects of radiotherapy may have had on this complication, particularly as many studies did not clearly state which incidences of haematoma were associated with the administration of radiotherapy, the effects of which have been discussed above.

Both types of mesh showed similar rates of increased complications in BR when radiotherapy was administered; however, ADMs may be more beneficial in this case, due to the apparent protective effect that has been discussed above. It could be argued that irradiation is an independent risk factor for complications in BR; outcomes in the literature investigating ADM use in the setting of radiation therapy are mixed.

Further limitations include variations between surgical techniques at both different sites and between different surgeons, which likely added noise to the data. In addition, the mastectomy type, type of mesh, type of reconstruction, and surgical technique were noticeably different between the studies. The lengths of follow-up period also varied considerably. Lastly, majority of the studies included were retrospective observational studies; therefore, it is unlikely that all the patient characteristics would have been adequately matched to rule out this bias.

## Conclusions

The overall consensus from the data presented shows that BR with synthetic matrices produces comparable aesthetic outcomes to ADMs, with lower costs and complication rates. However, in the absence of RCTs evaluating these outcomes, it is difficult to make any definite conclusions, particularly as the vast majority of published data on this topic looked at ADM use in BR. Further studies—particularly comparing the two types of mesh—are indicated, and a randomised controlled trial between synthetic and biological matrices is recommended by the authors. It could be argued that biological meshes are still leading the field at this point in time: their ability to incorporate into the host tissue, the small advantage they pose against capsular contracture, and their arguments for a protective effect against irradiation. In light of this, some might reason that these positives outweigh the negatives, and biological meshes could be considered over synthetic matrices in BR surgery.

## Additional file

Additional file 1: A complete table of all the studies compared. (DOCX 96 kb)

## Abbreviations

ADM: acellular dermal matrix
BR: breast reconstruction
CI: confidence interval
TE: tissue expander
