# Supplementary material for: Biological and synthetic mesh use in breast reconstructive surgery: a literature review
Source: World J Surg Oncol. 2016 Apr 21;14:121. doi: 10.1186/s12957-016-0874-9 (PMC4839154; doi:10.1186/s12957-016-0874-9)
Supplement: Additional file 1: — A complete table of all the studies compared. (DOCX 96 kb) [file 12957_2016_874_MOESM1_ESM.docx]

| Paper | Study method | Subject no. | Mastectomy type | Type of mesh | Type of reconstruction | Surgical technique | Results | Complications |
| --- | --- | --- | --- | --- | --- | --- | --- | --- |
| Tessler et al (2014)  Beyound biologic: absorable mesh as low-cost, low complication sling for implant-based breast reconstructions | Retrospective review | 50 patient (76 breasts) | Skin-preserving mastectomy | S = Vircyl mesh | Immediate | Direct-to-implant reconstruction = single stage | Mean follow up = 1.2 years  Excellent implant positioning and coutour with 2 patients (3 breast(3.9%)) undergoing revision procedures  Virycl mesh resultsed in direct cost savings of $172,112 | 5 breast (6.6%) complications = 1 complication resulting in implant loss |
| Becker & Lind (2013)  The use of synthetic mesh in reconstructive, revision, and cosmetic breast surgery | Retrospective review | 62 patient (112 breasts) |  | S = TIGR (R) matrix surgical Mesh | 11 primary reconstruction (19 breasts)  43 secondary/revision reconstruction (77 breasts)  3 augmentation/augmentation mastopexys (6 breasts) |  | Follow up = 9.4-26.2mnths  Average age = 54  Prior radiation = 9 patients (14.5%) | Postoperative breast complications:  Necrosis of two flaps = 1.8%  2 Seroma = 1.8%  4 infection/extrusiion = 3.6%  2 relapse of inflammatory fold/malposition = 1.8%  6 cases of asymmetry ( required corrective procedures |
| Dieterich et al (2013)  Implant-based breast reconstruction using a titanium-coated polypropylene mesh (TiLOOP Bra): a multicenter study of 231 cases | retrospective, multicenter, observational study | 207 ( 231 breasts) | Skin-sparing/nipple sparing mastectomy  or modified radical mastectomy | S = Titanium-coated polypropylene mesh (TiLOOP Bra) | Immediate reconstruction  Delayed reconstruction | Implant-based = single stage | Mean Follow up:? | Complications:  Majors were classified as those reconstructions that required addition surgery: infections, wound dehiscence, skin necrosis, seromas, and hematomas (13.4%)  Minors were those events that could be treated conservatively without surgical intervention (15.6%)  Implant loss (8.7%) |
| Selber et al (2013)  Autoderm: an alternative bioprosthetic for breast reconstruction | Retrospective study | 21 patients (36 breast) |  | B = Autoderm | Immediate | Tissue expander reconstructions (two stage) | Mean follow up = 1 year | Overall Complication (13.9%) occurred in 6 patients:  3 patients = mastectomy skin flap necrosis  1 = tissue expander exposure  No patients developed breast cellulitis, breast hyperaemia, infections or seroma |
| Rulli et al (2013)  Optimizing therapeutic timing in patients undergoing mastectomy through use of the Tiloop synthetic mesh: single-step surgery | Retrospective review | 4 patients (5 breasts) | Quadrantectomy (partial mastectomy)  Skin-sparing mastectomy  Nipple-sparing mastectomy | S = Tiloop synthetic mesh | Immediate | Implant-based reconstructions | no re-intervention  all patients underwent the required adjuvant therapy with no delay | mild erythema in the sin overlying the mesh but regressed after 20 days  no cases of super-infection or rejection |
| Kim & Cho (2013)  The suitability of absorbable mesh insertion for oncoplastic breast surgery in patients with breast cancer scheduled to be irradiated | Retrospective review | 35 patients | ? | Polyglactin vicryl mesh | Immediate | Implant-based reconstruction (One- stage) | Follow up = 23 months  91% cases there was an excellent/ good cosmetic outcome & 8.6% cases were fair before initiation of radiotherapy  After 6mnths and radiotherapy – there was a decrease in excellent or good (60%) and increase in fair outcomes (35.7%)  After 1 year, good cosmetic outcomes decreased from 65.7% to 42.9% and fair to poor increased from 8.6% to 27.1%  Significant factor affecting cosmetic outcome were pathology, specimen volume, and the estimated percentage of breast volume excised |  |
| Ibrahim et al (2013)  Acellular dermal matrices in breast surgery: a comprehensive review | Retrospective review | 26 series/studies review |  | Comparison of most common acellular dermal matrices used in breast surgery | Immediate and delayed breast reconstruction and others |  | Strattice + Permacol: highest max load sustained, stiffness, tensile strength |  |
| Clemens and Kronowitz (2012) Acellular dermal matrix in irradiated tissue expander/implant-based breast reconstruction: evidence-based review | Review + retrospective study | 276 patients |  | B: Acellular dermal matrix | Immediate breast reconstruction | Implant-based breast reconstruction | The use of acelluler dermal matrix in implant-based breast reconstruction in the setting of radiation therapy did not predispose to higher overall complication rates or prevent bioprosthetic mesh incorporation.  It also allowed an increase in intraoperative saline volume, which imporved aesthetic outcomes  But the rate of mesh incorporation may be slowed. |  |
| Dieterich et al (2012)  A short-term follow up of implant based breast reconstruction using a titanium coated polypropylene mesh | Retrospective review | 42 patients (45 breasts) | modified radical mastectomies  Skin-sparing mastectomy | S= titanium coated polypropylene mesh (TCPM) | Immediate and delayed breast reconstruction | Expander or implant-based breast reconstruction | Mean follow up = 26 months | 2 patients had mild hematoma, seroma/ infection  1 patient had skin necrosis or capsular contraction  3 patients required mesh explanation  These events were higher among the first cases with postoperative skin infection (p=0.003) |
| Dieterich et al (2012)  Using a titanium-coasted polypropylene mesh for implant-based breast reconstruction: case report and histological analysis | Case report and histological analysis | 1 patient | Skin-sparing mastectomy | S= titanium coated polypropylene mesh (TCPM) | Immediate | Tissue expander ( 2-stage) | fibers of the mesh were well embedded into collagen-rich fibrillary scar tissue. Only a mild infiltration  with inflammatory cells was observed,  indicating a good biocompatibility |  |
| Singh et al (2012)  Immediate-1 stage vs tissue expander postmastectomy implant breast reconstructions: a retrospective real-world comparison over 18 months | Retrospective review | 1,316 patients |  | S= titanium coated polypropylene mesh (TCPM) | Immediate | Implant-based breast reconstruction = one stage  or  tissue expander based reconstruction = two stage | Non significant trend towards fewer returns visits after Immediate-1 stage (28.4%) vs tissue expander (27.4%) | Complication of the implant, graft/mesh were the most common complications experienced by 28.4% of 1-stage and 27.4% of TE reconstruction  Complications involing the skin and connective tissue were also common with 20.0% in 1-stage and 26.4% in TE |
| Adetayo et al (2011)  A meta-analysis of outcomes using aceelular dermal matrix in breast and abdominal wall reconstructions : events rates and risk factors predictive of complications | Meta-analysis | 53 articles |  | B = Acellular dermal matrix |  |  | Mean follow up for breast = 14.2 months | Results for breast:  6.1% implant failure  5.1% wound infection  4.4% cellulitis  4.1 seroma  2% wound dehiscence  (association of complication with risk factors is grouped for breast + abdo so N/A) |
| Nyame et al (2011)  High-throughput assay for bacterial adhesion on acellular dermal matrices and synthetic surgical material | ? |  |  | 2 synthetic:  Prolene  Vicryl  2 biologic:  Alloderm  FlexHD |  |  | S.aureus adheres more readily to acellular dermal matrix than to synthetic mesh |  |
| Loustau et al (2007)  Immediate prosthetic breast reconstruction: the ensured subpectoral pocket (ESP) | Observational study | 24 patients | Total mastectomy | S = polyglycolic mesh | I | 1-Stage | Mean Follow time = 2.8 years  All breast reconstruction successful | No capsular contracture, infection, local recurrence  Good symmetry + pt satisfaction  1 unilat haematoma  1 seroma  2 partial wound dehiscence |
| Rietjens et al (2005)  The suspension technique to avoid the use of tissue expanders in breast reconstruction | Observational study | 73 patients | radical modified mastectomy  total mastectomy | S = Non-abosorable Mersilene | I + D |  | Mean follow up =  56 pts (76.7%): no further surgery with general anesthesia  14 pts (19.2%) second operation was required for implant replacement, capsula revision, and nipple and aerola (NAC) RECONSTRUTION  3 Pts ( 4.1%) implant removal was necessary due to implant exposition or infection  50 capsular contracture  Breast symmetry (7.56%)  Pts satisfaction (7.75%)  Surgeon’s cosmetic evaluation (7.60%) |  |
| Bank et al (2013)  Economic analysis and review of the literature on implant-based breast reconstruction with and without the use of the acellular dermal matrix | Retrospective review | 132 patients |  | B = Alloderm (61)  Strattice (23) | Immediate | 2-stage | No. Of fills required to achieve final volume was higher in the non-ADM group than in the ADM group (p<0.0001)  No sig. Diff in the small volume fill (P>0.05)  Sig diff found in the higher volume fill  (p<0.05)  Therefore ADM in 2-stage recon reduces the no. Of visits for recon with 350ml or more. But the cost of ADM is not offset by the reduce no. Of visits. n |  |
| Fahrenbach et al (2013)  Resistance of acellular dermal matrix material to microbial penetration | Comparative study |  |  | B=  AlloDerm  FlexHD  Strattice  NeoForm |  |  | AlloDerm &  FlexHD had the best barrier to penetration to S. Pyogenes  AlloDerm & FlexHD & Strattice prevented penetration by S.aureus & S. Pyogenes  NeoForm was less effective in withstanding the organisms  4 Biological mesh were resistant to in vitro penetration by S.aureus, S. Pyogenes and partially resistant to P.aeruginosa.  Resistance to fungal pathogen is uncertain |  |
| Spear et al (2013)  Porcine acellular dermal matrix (strattice) in primary and revision cosmetic breast surgery | Retrospective review | 43 patients (75 breasts) |  | Strattice | Primary and revision cosmetic surgery | The use of Strattice is safe and be helpful in the management of certain situation in cosmetic breast surgery | Mean follow up = 17.5 months  64 of 75 patients (98.7%) had successfully achieved the indication for which the Strattice was placed  1 breast had some degree of relapse (1.2%)  Global complication rate was 5.3%:  Postoperative malposition (2.7%)  Bottoming up (1.3%)  Infection (1.2%) |  |
| Johnson et al (2013)  Cost minimisation analysis of using acellular dermal matrix (strattice) for breast reconstruction with standard technique | Retrospective review | 24 patients |  | Strattice | Immediate | Implant = one-stage | Unilateral strattice (£3685)  Unilateral TE (£4985)  Unilateral LD and implant (£6321)  Bilateral TE (£5478)  Bilateral Strattice (£6771) |  |
| Salzberg et al (2013)  Immediate breast reconstruction using porcine acellular dermal matrix (strattice): long-term outcomes and complications | Retrospective study? | 54 patients (105 reconstructions) |  | Strattice | Immediate breast reconstruction | One-stage (101 reconstructions)    &  Two stage (4 reconstructions) | Mean follow up = 3.5 years | Complication in 9 breasts:  Implant loss or explanation (3.8%)  Infection (3.8%)  Skin breakdown or necrosis (2.9%)  Seroma (1.9%)  Implant exposure (1.0% )  Delayed skin delayed (1.0%)  Histological analyses of implanted strattice revealed a viable matrix with fibroblast infiltration and revascularization |
| Gandhi et al (2013)  Bioprosthetics: changing the landscape for breast reconstruction? | Review on breast reconstruction? |  |  |  |  |  |  |  |
| Hester et al (2012)  Use of dermal matrix to prevent capsular contracture in aesthetic breast surgery | Retrospective study? | 80 patients  ( 154 breast) |  | Strattice | Not for reconstruction | Revision in patients with capsular contracture or  Primary augmentation or augmentation mastopexy | Data shows that Strattice has significantly lower rate of capsular contracture in the first 3.5 years after implant placement | Clinically significant contracture occurred in 3 patients (3.75%)  2 seroma required implant removal  2 haematomas required revision  Overall failure rate of 6.25% for Strattice-assisted surgery |
| Abood and Rhodes et al?  A simple technique to enhance breast aesthetics using porcine acellular dermal matrix (Strattice) in breast reconstruction |  |  |  |  |  |  |  |  |
| Galsberg & Light  AlloDerm and Strattice in breast reconstruction: a comparison and technique for optimizing outcomes | Retrospective study | 186 patients (269 reconstructions) |  | B =  Strattice (126 reconstruction)  Allorderm (144 reconstructions) | Immediate | two-stage breast reconstruction | Complication in this study were of low severity | Total complication were significantly higher with Alloderm (21.4%) reconstruction  Seroma complication were significantly higher in alloderm (12.7%) P=0.0003  All other complications were similar between the groups  Capsular contraction was similar in alloderm (2.4%) and Strattice group (2.8%) indicating that both play a role in capsule formation |
| Cheng & Saint  Comparison of different ADM materials in breast surgery | Literature review? |  |  |  |  |  |  |  |
| Israeli et al (2010)  Accelular dermal matrix in breast reconstruction in the setting of radiotherapy | Literature review? |  |  |  |  |  |  |  |
| Krishnan et al  The cost effectiveness of acellular dermal matrix in expander-implant immediate breast reconstruction | Retrospective review |  |  | B = Alloderm | I | two-stage | The decision model revealed a baseline cost increase of $361.96 when acellular dermal matrix is used. The increase in Quality-Adjusted Life Years (QALYs) is 1.37 in the population with acellular dermal matrix. This yields a cost effective incremental cost-utility ratio (ICUR) of $264.20/QALY. Univariate sensitivity analysis confirmed that using acellular dermal matrix is cost effective even when using retail costs for unilateral and bilateral reconstructions. | The overall complication rates were 30% and 34.5% with and without ADM. |
| Moyer et al (2014)  The effect of radiation on acellular dermal matrix and capsule formation in breast reconstruction: clinical outcomes and histologic analysis. |  | 27 patients |  |  |  |  | mean follow-up was 28 months  Acellular dermal matrix appears to limit the elastosis and chronic inflammation seen in irradiated implant reconstructions and is potentially beneficial in these patients | Grade III/IV contractures were identified in nine patients (all on the irradiated side), and 12 developed noncontracture complications (75 percent on the irradiated side). Nine patients were unable to continue with implant reconstruction and required salvage with autologous tissue. In part II, postirradiation biopsy specimens were taken of the peri-implant capsule in six patients at the time of secondary surgery. Elastin content and the total cellular infiltrate were significantly greater in the irradiated versus nonirradiated native capsules (p = 0.0015). Conversely, the irradiated matrix capsule was composed of similar amounts of cellular infiltrate and collagen as the nonirradiated matrix capsules and nonirradiated native capsules. Irradiated acellular dermal matrix showed the least amount of alpha-smooth actin staining but a similar number of blood vessels. |
| Forsberg et al (2013)  Aesthetic Outcomes of Acellular Dermal Matrix in Tissue Expander/Implant-Based Breast Reconstruction. | retrospective chart review | 122 patients underwent 183 tissue expander-based reconstructions |  |  | I | 2-stage | These consistent findings suggest that the use of ADM in breast reconstruction does confer a significant advantage in aesthetic outcomes for breast reconstruction. This is likely at the cost of a higher infection rate when using ADM; however, that may be offset by the advantage of a lower rate of capsular contracture in patients with ADM. | The infection rate in patients with ADM was 16.2% compared to 5.9% in TSR patients, but this was not statistically significant (P = 0.09). Capsular contracture was more common in TSR patients (23.5%), compared to those with ADM (8.1%), P = 0.048. Aesthetic scores from the attending plastic surgeons were as follows: natural contour (ADM, 3.36; TSR, 3.02; P = 0.0001), symmetry of shape (ADM, 3.57; TSR, 3.27; P = 0.005), symmetry of size (ADM, 3.68; TSR, 3.42; P = 0.002), position on chest wall (ADM, 3.75; TSR, 3.45; P = 0.004), and overall aesthetic appearance (ADM, 3.56; TSR, 3.20; P = 0.0001). |
| Ibrahim et al (2013)  Analysis of the National Surgical Quality Improvement Program database in 19,100 patients undergoing implant-based breast reconstruction: complication rates with acellular dermal matrix. | retrospective review | 19,100 cases, 3301 involved acellular dermal matrix use |  | B= acellular dermal matrix use |  |  |  | Overall complication rates were not statistically significant (acellular dermal matrix, 5.3 percent; non-acellular dermal matrix, 4.9 percent; p=0.396). Several risk factors were statistically significant associated factors of complications. Higher body mass index was associated with wound complications in both cohorts. In the non-acellular dermal matrix group, body mass index, smoking, and diabetes were associated with major complications, and radiotherapy and steroid use with minor complications. |
| Ganske et al. (2013)  Minimizing complications with the use of acellular dermal matrix for immediate implant-based breast reconstruction. | Retrospective observational | 179 breasts: 106 w ADM vs 73 w/o |  | B: acellular dermal matrix vs without | I | 2-stage | Follow-up: 18m | Overall native breast skin flap necrosis rate was significantly higher in the ADM group (28.3% vs 5.5%, P = 0.0003). No significant differences in the rate of seroma, infection, or skin flap necrosis between 2-stage tissue expander reconstruction and the single-stage implant reconstruction.  Compared to 150 pts (published data): 64 w ADM, 86 w/o. This study had modification in management. Statistically significant reduction in the rates of seroma and major infection (4.7% vs 18.6% rate of seroma, and 1.9% vs 7% rate of major infection. No diff in skin flap necrosis. |
| Heyer et al (2010)  Reversed acellular dermis: failure of graft incorporation in primary tissue expander breast reconstruction resulting in recurrent breast cellulitis. | Retrospective review | 1 patient (1 breast) | skin-sparing mastectomy | B= Flex-HD | I | 2-stage |  | There were no immediate postoperative complications. While receiving adjuvant chemotherapy, she had multiple episodes of self-limiting superficial cellulitis responsive to oral antibiotics. Ultrasound imaging revealed no evidence of fluid collection or abscess.  At the routine expander/implant exchange procedure, intraoperative examination revealed capsule formation surrounding the acellular dermis graft, without significant tissue incorporation. On closer evaluation, the graft was noted to be reversed, with the tissue ingrowth dermal surface facing toward the expander. The unincorporated graft was removed. Postoperatively, the patient did not experience any further episodes of cellulitis. Pathologic evaluation of the dermal graft revealed |
| Spear& Scott (2009)  Discussion: Acellular Dermis–Assisted Prosthetic Breast Reconstruction versus Complete Submuscular Coverage: A Head-to-Head Comparison of Outcomes | retrospective review with randomisation | 100 patients 50w ADM vs 50 w/o |  | B: acellular dermal matrix vs without | I | 2-stage | At the routine expander/implant exchange procedure, intraoperative examination revealed capsule formation surrounding the acellular dermis graft, without significant tissue incorporation. On closer evaluation, the graft was noted to be reversed, with the tissue ingrowth dermal surface facing toward the expander. The unincorporated graft was removed. Postoperatively, the patient did not experience any further episodes of cellulitis. Pathologic evaluation of the dermal graft revealed dense foreign-body reaction without evidence of tissue revascularization | There was no significant difference in total complication rate between the two groups, and this included no difference in terms of rates of seroma, breast cellulitis, or infection. Three infections required expander removal in the submuscular group, and four infections required expansion removal in the acellular dermis–assisted group. The findings of this study confirm previous beliefs about two-stage expander/implant reconstruction: the procedure is effective and safe and, although it can be achieved using total submuscular placement, can be performed more rapidly using acellular dermis |
| Sbitany et al (2009)  Acellular dermis-assisted prosthetic breast reconstruction versus complete submuscular coverage: a head-to-head comparison of outcomes. | retrospective review | 100 patients (172 recons)  50w ADM vs 50 w/o |  | B: acellular dermal (AlloDerm) matrix vs without | I | 2-stage | The patient groups were similar in terms of demographic data. Mean number of fills to complete reconstruction was 4.31 in the submuscular group and 1.72 in the acellular dermis group (p = 0.0001). Mean intraoperative fill volume was 130 cc in the submuscular group, compared with 412 cc per expander in the acellular dermis group (p = 0.0001). Fisher's exact test demonstrated no significant difference in total complication rate between the two groups (14 percent versus 18 percent; p = 0.79). | Acellular dermis allowed for a greater initial fill of saline. This potentially improves cosmetic outcome, as it better capitalizes on preserved mastectomy skin for reconstruction. The authors conclude that acellular dermis-assisted implant breast reconstruction has a safety profile no worse than that of complete submuscular coverage but offers the benefit of fewer expansions and the potential for more predictable secondary revisions. |
| Antony et al (2010)  Acellular Human Dermis Implantation in 153 Immediate Two-Stage Tissue Expander Breast Reconstructions: Determining the Incidence and Significant Predictors of Complications | retrospective review | 96 patients 153 expanders were implanted |  | B: AlloDerm | I | 2-stage | Univariate analysis revealed age, body mass index, axillary dissection, and postoperative chemotherapy to be associated with reconstructive failure (p < 0.05). Multivariate analysis revealed that age, body mass index, and axillary dissection are independent risk factors for developing complications (p < 0.05). | Eleven (7.2 percent) were removed due to infection (n = 5, 3.3 percent), exposure (n = 4, 2.6 percent), or patient preference (n = 2, 1.3 percent). Other complications included cellulitis (3.9 percent), seroma (7.2 percent), hematoma (2.0 percent), mastectomy flap necrosis (4.6 percent), and leak/failed expansion (0.0 percent); 92.8 percent were successfully expanded and exchanged for a permanent implant. Eleven seromas (7.2 percent) were identified; nine underwent aspiration. None of these resulted in infection or reconstructive failure. |
| Newman et al (2011)  The true incidence of near-term postoperative complications in prosthetic breast reconstruction utilizing human acellular dermal matrices: a meta-analysis. | Meta-analysis | 789 breasts |  | B:  Human acellular dermal matrix  AlloDerm  Flex HD_  NeoForm_  DermaMatrix |  |  | mean follow-up was 13.7 months | Under the random-effects model, the total complication rate was 12.0%. The most common complications were flap necrosis (3.3%), seroma (3.3%), and infection (5.6%). All complications not included in these categories were set apart in a separate category, "Other," and totaled 3.0% |
| Sbitany et al (2011)  [Acellular dermal matrix in primary breast reconstruction.](http://www.ncbi.nlm.nih.gov/pubmed/21908822) | Literature review (read) |  |  |  |  |  |  |  |
| Hanna et al (2013)  Comparison study of two types of expander-based breast reconstruction: acellular dermal matrix-assisted versus total submuscular placement. | retrospective review | 75 patients 100 breast reconstructions  31 with ADM and 44 with a submuscular coverage technique |  | B: acellular dermal matrix | I | 2-stage | Mean follow-up time from stage II to the last visit was 9.6 and 7.7 months for submuscular and ADM-assisted groups, respectively | Total complications including seroma, hematoma, infection, skin necrosis, and explantation did not significantly differ between groups (n = 13 for ADM vs. 17 for submuscular, P = 0.814). Consistent with prior reports, ADM-based reconstructions were associated with significantly increased intraoperative fill volumes and lower total number of sessions to achieve final volume. Submuscular reconstructions required a significantly higher tissue expander fill volume. Eight patients in the submuscular group required surgical revision of the breast and inframammary fold, compared with 4 in the ADM group; however, this difference was not significant. Patient satisfaction was equivalent between the 2 groups; however, it was higher in patients with bilateral reconstruction and lower among those who had received adjuvant radiation therapy. |
| Collis et al (2012)  Acellular dermal matrix slings in tissue expander breast reconstruction: are there substantial benefits? | retrospective review | 63 patients (106 breasts) in the ADM group and 42 patients (68 breasts) in the control group |  | B: acellular dermal matrix | I | 2-stage | Initial intraoperative fill volumes were significantly greater in the ADM group, median 69% full (250 mL) versus 50% full (180 mL; P < 0.001). However, the number of days to complete expansion between the 2 groups was similar. One less office visit was required to complete the fills in the ADM group (P < 0.01). Drains were removed 3 days later in the ADM group (P < 0.01). | Overall complication rate was greater in the ADM group (18.9% vs. 7.4%, P < 0.05), with a slightly higher percentage of expanders requiring removal due to infection in the ADM group (5.7% vs. 4.4%, P = NS). This study suggests inferolateral ADM slings in expander-based breast reconstruction allow for significantly increased initial fill volumes and may offer an aesthetic advantage; however, its use is costly and increases complications. |
| Cassileth et al (2012)  One-stage immediate breast reconstruction with implants: a new option for immediate reconstruction. | retrospective review | 43 sequential patients on a total of 78 breasts |  | B: acellular dermal matrix | I | One-stage | Follow-up was for an average of 575 days | Complication rates were as follows: seroma occurred in 6.4% of breasts; infection resolving with antibiotics occurred in 2.6%; infection requiring implant removal occurred in 3.8%; and hematoma occurred in 1.3%. Neither preoperative breast size nor implant size correlated to an increased risk of complications (P>0.05). Complication rate increased with age (P=0.02). The average score for the preoperative images was 2.1, whereas the postoperative average was 2.4. This represented a statistically significant improvement above the baseline (preoperative) breasts with a P<0.001, according to a 2-sided paired t test. |
| de Blacam et al (2012)  Cost analysis of implant-based breast reconstruction with acellular dermal matrix. |  |  |  |  |  |  | The most expensive procedure at baseline was TE/I + ADM ($11,255.78), followed by TE/I alone ($10,934.18), and SSI + ADM ($5,423.02). Incorporating the probability of complications as derived from the published literature into the cost analysis resulted in an increase in the excess cost of ADM-based procedures (TE/I + ADM, $11,829.02; TE/I, $11,238.60; SSI + ADM, $5,909.83). Although SSI + ADM have the lowest cost, not all patients are suitable candidates for this type of procedure. With increasing focus on healthcare expenditure, it is important that plastic surgeons are aware of the cost implications of using ADM products. |  |
| Ho et al (2012)  A systematic review and meta-analysis of complications associated with acellular dermal matrix-assisted breast reconstruction. | systematic review and meta-analysis |  |  |  |  |  |  | The pooled complication rates were seroma (6.9%; 95% CI, 5.3%-8.8%), cellulitis (2.0%; 95% CI, 1.2%-3.1%), infection (5.7%; 95% CI, 4.3%-7.3%), skin flap necrosis (10.9%; 95% CI, 8.7%-13.5%), hematoma (1.3%; 95% CI, 0.6%-2.4%), capsular contracture (0.6%; 95% CI, 0.1%-1.7%), and reconstructive failure (5.1%; 95% CI, 3.8%-6.7%). Five studies reported findings for both the ADM and non-ADM patients and were used in the meta-analysis to calculate pooled OR. ADM-assisted breast reconstructions had a higher likelihood of seroma (pooled OR, 3.9; 95% CI, 2.4-6.2), infection (pooled OR, 2.7; 95% CI, 1.1-6.4), and reconstructive failure (pooled OR, 3.0; 95% CI, 1.3-6.8) than breast reconstructions without the use of ADM. The relation of ADM use to hematoma (pooled OR, 2.0; 95% CI, 0.8-5.2), cellulitis (pooled OR, 2.0; 95% CI, 0.9-4.3), and skin flap necrosis (pooled OR, 1.9; 95% CI, 0.6-5.4) was inconclusive. |
| Endress et al (2012)  Use of fetal bovine acellular dermal xenograft with tissue expansion for staged breast reconstruction. | retrospective review | 49 breast reconstructions in 28 patients (group A) with FBADM were retrospectively compared with 123 reconstructions in 91 patients operated without FBADM |  | B:  SurgiMend | I | 2-stage | The mean immediate fill volume in group A was 181.2 ± 148.3 mL and 117.7 ± 66.3 mL in group B (P < 0.001). The duration of drainage was significantly shorter in group A (8.51 ± 3.4 days) as compared with controls (11.07 ± 5.1 days), t-test (P = 0.015). | There was no significant difference in the overall complication rate (20.8% in group A, 13.0% in group B). Further subgroup analysis of group A patients with complications and without complications, showed that group with complications had significantly longer drain removal time (9.48 vs. 7.97 days), larger initial fill volumes (238.1 vs. 145.3 mL), and a higher BMI (25.8 vs. 22.6 kg/m2) when compared with the complication-free subgroup. |
| [T. JoAnna Nguyen](http://www.sciencedirect.com/science/article/pii/S174868151100043X) et al (2011)  Use of human acellular dermal matrix in implant- based breast reconstruction: Evaluating the evidence | Review Of ADM (read good structure) |  |  |  |  |  |  |  |
| Himsl et al (2012) The use of porcine acellular dermal matrix in silicone implant-based breast reconstruction. | Retrospective review | 23 patients (27 breasts |  | B=  Strattice |  | 1-stage | 18 (78%) were "satisfied" with the aesthetic and haptic outcome after implant-based reconstruction with PADM. One patient (one breast) required another breast operation because of ipsilateral breast cancer recurrence during the follow-up period. PADM-assisted implant-based breast reconstruction has a satisfactory safety profile |  |
| [Kobraei EM](http://www.ncbi.nlm.nih.gov/pubmed?term=Kobraei%20EM%5BAuthor%5D&cauthor=true&cauthor_uid=22286439) et al (2012)  Risk factors for adverse outcome following skin-sparing mastectomy and immediate prosthetic reconstruction. | Retrospective review | 102 patients (155 breasts | skin-sparing mastectomy | B= AlloDerm | I | One stage and two stage |  | The use of acellular dermis was associated with a greater than three-fold increased risk of postoperative complications. Radiotherapy exposure was found to have a significant association with implant loss. None of the patient-related characteristics studied behaved as risk factors for postoperative complications, and none of the procedure-related characteristics acted as risk factors for implant loss. |
| Kim et al (2012)  A meta-analysis of human acellular dermis and submuscular tissue expander breast reconstruction. | meta-analysis | 48 relevant studies |  |  |  |  |  | Nineteen studies reporting human acellular dermal matrix (n = 2037) and 35 reporting submuscular outcomes (n = 12,847) were used to estimate complication rates. Rates were generally higher in acellular dermis patients: total complications, 15.4 versus 14.0 percent; seroma, 4.8 versus 3.5 percent; infection, 5.3 versus 4.7 percent; and flap necrosis, 6.9 versus 4.9 percent. Six studies reporting both acellular dermis and submuscular outcomes were used to estimate relative risks. There was an increased risk of total complications (relative risk, 2.05; 95 percent CI, 1.55 to 2.70), seroma (relative risk, 2.73; 95 percent CI, 1.67 to 4.46), infection (relative risk, 2.47; 95 percent CI, 1.71 to 3.57), and reconstructive failure (relative risk, 2.80; 95 percent CI, 1.76 to 4.45) in acellular dermis patients. |
| Gabriel et al (2011)  Evolving Role of AlloDerm in Breast Surgery | Good review Read |  |  |  |  |  |  |  |
| Colwell et al (2011)  Retrospective review of 331 consecutive immediate single-stage implant reconstructions with acellular dermal matrix: indications, complications, trends, and costs. | Retrospective review | 207 (331 breast) | nipple-sparing (n = 66)  skin-sparing (n = 265) mastectomy | B= AlloDerm | I | 1- stage |  | Fifty-one patients underwent preoperative (n = 33) or postoperative (n = 18) irradiation. Total complications included 10 infections (3.0 percent), five seromas (1.5 percent), four hematomas (1.2 percent), and 30 reconstructions (9.1 percent), with skin necrosis leading to five implant losses (1.5 percent). Tissue expander reconstruction without AlloDerm had a similar total complication rate (158 reconstructions) (p = 0.18), including nine infections (5.7 percent), three seromas (1.9 percent), three hematomas (1.9 percent), and 16 reconstructions (10.1 percent), with skin necrosis leading to 11 implant losses (7.0 percent). A higher complication rate occurred in the surgeons' combined first year performing single-stage implant reconstruction (21.4 percent) compared with subsequent years (10.9 percent) (p < 0.02) and in one- or two-stage reconstruction patients undergoing irradiation (p = 0.005). There was no significant difference in total overall costs (p = 0.8). |
| Sbitany and Serletti (2011)  Acellular dermis-assisted prosthetic breast reconstruction: a systematic and critical review of efficacy and associated morbidity. | systematic and critical review | 6 studies |  | B= AlloDerm  Strattice  FlexHD |  |  |  | The only difference found in complications was a higher rate of seroma for the acellular dermal matrix group (4.3 percent versus 8.4 percent, p = 0.03). Despite this, both groups illustrated similar rates of infection leading to explantation (3.2 percent for submuscular and 3.4 percent for acellular dermal matrix, p = 0.18). In addition, acellular dermal matrix techniques illustrated greater intraoperative fill volumes and consistently fewer fills required to reach expander capacity. |
| [Hoppe](http://www.ncbi.nlm.nih.gov/pubmed?term=Hoppe%20IC%5BAuthor%5D&cauthor=true&cauthor_uid=22084645) et al (2011)  Complications following expander/implant breast reconstruction utilizing acellular dermal matrix: a systematic review and meta-analysis. | systematic review and meta-analysis | 8 studies |  | B= AlloDerm |  |  |  | There was more than a 2-fold increase in the number of infections and explanations in the acellular dermal matrix group compared to the control. There was a 3-fold increase in seroma formation in the acellular dermal matrix group compared to the control. There was a significant difference of intraoperative fill volumes between the acellular dermal matrix group compared to the control |
| [Vardanian AJ](http://www.ncbi.nlm.nih.gov/pubmed?term=Vardanian%20AJ%5BAuthor%5D&cauthor=true&cauthor_uid=22030500) et al (2011)  Comparison of implant-based immediate breast reconstruction with and without acellular dermal matrix. | Retrospective review | 203 patients (337 reconstruction)  with ADM n=208 (61.7%); without, n=129 (38.3%) |  | B= AlloDerm | I | 2-stage | Mean follow up:? | Complications occurred in one-third of patients (33.5 percent). In univariate analyses, acellular dermal matrix use had fewer overall complications (odds ratio, 0.61; 95 percent CI, 0.38 to 0.97). The incidences of seroma/hematoma (p=0.59), infection (p=0.31), and wound complications (p=0.26) did not differ. Aesthetic outcomes were higher in the acellular dermal matrix group. In multivariate logistic regression, acellular dermal matrix use was associated with less capsular contracture (odds ratio, 0.18; 95 percent CI, 0.08 to 0.43) and mechanical shift (odds ratio, 0.23; 95 percent CI, 0.06 to 0.78). |
| Seth et al (2012)  Utility of acellular dermis-assisted breast reconstruction in the setting of radiation: a comparative analysis. | Retrospective review | 417 patients (592 breasts)  with ADM n= 199  without, n= 393 | skin-sparing or modified skin-sparing mastectomy | B= AlloDerm | I | 2-stage | Similar Mean follow-up for ADM and non-ADM patients was 23.2 ± 8.9 months and 24.4 ± 12.7 months  Breasts with acellular dermis had larger intraoperative fill volumes (p < 0.0001) and decreased postoperative expansions (p = 0.02), but no decrease in time to implant exchange. | There were no significant differences in complication profiles between acellular dermis and non-acellular dermis breasts, after adjusting for other relevant patient variables on regression analysis. After stratifying patients by exposure to radiation, acellular dermis breasts had a decreased risk of all complications related to radiation as compared with non-acellular dermis breasts. |
| Slavin, Sumner A (2012)  Discussion: Human Acellular Dermis versus No Acellular Dermis in Tissue Expansion Breast Reconstruction | Discussion Read |  |  |  |  |  |  |  |
| [Parks et](http://www.ncbi.nlm.nih.gov/pubmed?term=Parks%20JW%5BAuthor%5D&cauthor=true&cauthor_uid=23018685) al (2012)  Human acellular dermis versus no acellular dermis in tissue expansion breast reconstruction. | retrospective review | 232 patients (346 breasts) |  | B = AlloDerm | I | 2-stage | Mean follow up =? | Seroma occurrence in the acellular dermis group was nearly twice (30.0 versus 15.1 percent) that of the no acellular dermis breasts, but the tissue expander loss was only slightly higher (11.6 versus 8.5 percent) and not statistically significant. Body mass index in patients who lost their tissue expander was 31 kg/m, statistically significantly higher than in those who did not. |
| Venturi et al (2013)  Evaluating sterile human acellular dermal matrix in immediate expander-based breast reconstruction: a multicenter, prospective, cohort study. | multicenter, prospective, cohort study | 39 patients (65 reconstructions) |  | B=  AlloMax | I | 2-stage | Mean follow up =? | Complications occurred in three breasts (4.6 percent), including one case of cellulitis (1.5 percent) and two cases of partial mastectomy flap necrosis (3.0 percent) that required débridement. There were no seromas or explantations. The grafts were incorporated in all cases and verified histologically in the first 20 biopsies |
| Larcher et al (2012)  Acellular Dermis–Assisted Prosthetic Breast Reconstruction: Mission Accomplished? | Review – read |  |  |  |  |  |  |  |
| Brooke et al (2012)  Complications in tissue expander breast reconstruction: a comparison of AlloDerm, DermaMatrix, and FlexHD acellular inferior pole dermal slings. | retrospective analysis | 173 patients (284 reconstruction)  49=AlloDerm  110 =DM,  62 =FHD,  64=ADM |  | B= AlloDerm, DermaMatrix (DM), FlexHD (FHD | I | 2 stage | Mean follow up=? | The total complication rate with AlloDerm was 22% [95% confidence interval (CI), 11-34], with DM was 15% (95% CI, 8-21), and with FHD was 18% (95% CI, 8-28) (P=0.47). Infectious complication rates for AlloDerm, DM, and FHD were equal at 10% (P=0.97). The total complication rate of all ADM reconstructions as a grouped cohort was 17% compared to 11% without ADM (P=0.48). The overall incidence of infectious complications with ADM was 10% compared to 2% without ADM (P=0.09). There is no difference in the clinically significant overall complication rate or incidence of infection between AlloDerm, DM, and FHD. Isolating infectious complications, there is a trend toward increased incidence with ADM compared to reconstructions without. |
| Bonomi et al (2012)  Implant-based breast reconstruction with acellular dermal matrix. | Review read |  |  |  |  |  |  |  |
| Spear et al 2012)  Two-stage prosthetic breast reconstruction using AlloDerm including outcomes of different timings of radiotherapy. | Retrospective review | 289 women (428 breasts |  | B= AlloDerm | I | Two-stage |  | After first-stage reconstruction, clinically significant capsular contracture rates (grade III/IV) were higher in the radiation therapy during expansion group and in the radiation therapy before mastectomy group compared with the no-radiation therapy group. Three hundred fifty-three breasts (85.9 percent) successfully underwent second-stage reconstruction, with a median follow-up of 15.2 months. Of those 353 breasts, clinically significant capsular contracture (grade III/IV) was highest in the radiation therapy during expansion group. More often than in the other groups, the radiation therapy during expansion group failed two-stage reconstruction and required flaps in addition or as replacement. |
| Peled et al (2012) The effects of acellular dermal matrix in expander-implant breast reconstruction after total skin-sparing mastectomy: results of a prospective practice improvement study | Prospective review | 3 groups of patients  No ADM = 90 cases  With ADM = 100 cases  Selective ADM =260 cases | total skin-sparing mastectomy | B=AlloDerm | I | 2-STAGE | Mean follow-up was 25.5 months | Infection occurred in 27.8 percent of the no-acellular dermal matrix cases, 20 percent of the consecutive cases, and 15.8 percent of the selective cases (p = 0.04). Unplanned return to the operating room was required in 23.3, 11, and 10 percent of cases, respectively (p = 0.004). Expander-implant loss occurred in 17.8, 7, and 5 percent of cases, respectively (p = 0.001). Additional analysis of the odds ratios of developing complications after postmastectomy radiation therapy demonstrated a specific protective benefit of acellular dermal matrix in irradiated patients |
| Butler and Selber (2012)  Discussion: The Use of Acellular Dermal Matrix in Immediate Two-Stage Tissue Expander Breast Reconstruction | Discussion read |  |  |  |  |  |  |  |
| Weichman et al (2012)  The use of acellular dermal matrix in immediate two-stage tissue expander breast reconstruction. | retrospective analysis | 407 patients (628 reconstrution)  442 With ADM  186 Without ADM |  | B= AlloDerm | I | 2-STAGE | Mean follow =? | however, major complications were significantly increased in the acellular dermal matrix group (15.3 versus 5.4 percent; p = 0.001). These complications included infection requiring intravenous antibiotics (8.6 versus 2.7 percent; p = 0.001), flap necrosis requiring excision (6.7 versus 2.7 percent; p = 0.015), and explantation of the tissue expander (7.7 versus 2.7 percent; p = 0.004). |
| Hill et al (2012)  Infectious complications associated with the use of acellular dermal matrix in implant-based bilateral breast reconstruction. |  | 79 patients |  | B=ADM? |  | 1-stage |  | On univariate analysis, the use of ADM (31% vs. 7%, P = 0.018), smoking (37% vs. 13%, P = 0.045), and open wound (55% vs. 13%, P = 0.006) were significantly associated with increased risk of infection. Multivariate analysis revealed open wound as the strongest predictor of infection. |
| McCarthy et al (2012)  Discussion: Utility of Acellular Dermis–Assisted Breast Reconstruction in the Setting of Radiation: A Comparative Analysis | Discussion: read |  |  |  |  |  |  |  |
| Kim and Connor 2012  Focus on Technique: Two-Stage Implant-Based Breast Reconstruction | Read |  |  |  |  |  |  |  |
| Cordeiro (2012)  Discussion: Focus on technique: two-stage implant-based breast reconstruction | Read |  |  |  |  |  |  |  |
| Becker et al. (2009)  AlloDerm vs DermaMatrix in immediate expander-based breast reconstruction... | Retrospective analysis | 30  (50 breasts) |  | B (AlloDerm vs DermaMatrix) | I + D | Expander-based extension of pec. maj. (2 stage) | Follow u: 6.7 months  Similar results: Both well incorporated with neovascularisation | Similar rates. Overall complication rate: 4%. 1 seroma and 1 infection/cellulitis in the DermaMatrix cohort. Cellulitis resolved with IV antibiotics + seroma resolved with a single needle aspiration. |
| Jansen et al (2011)  The use of AlloDerm in postmastectomy alloplastic breast reconstruction: part I | Systematic review | 14 studies |  | B: AlloDerm vs none | I (except a subset of pts in 2 studies) | Single stage/ Direct to implant vs 2-stage |  | Infection:0-4%; Haematoma:0-6.7%); Seroma:0-9%); Partial flap necrosis:0-25%); Implant exposure w removal:0-14%; Implant exposure w salvage:0-4%; Capsular contracture:0-8%); Rippling:0-6%) |
| Jansen et al (2011)  The use of AlloDerm in postmastectomy alloplastic breast reconstruction: part II | Cost analysis from recent systematic review |  |  | B: AlloDerm vs none | I (except a subset of pts in 2 studies) | Single stage/ Direct to implant vs 2-stage | Immediate/Direct-to-implant with AlloDerm less expensive at baseline + expected costs (incl. w same contracture rate) |  |
| Katerinaki et al. (2010)  Histological appearance of Strattice tissue matrix used in breast reconstruction. | Histological confirmation of integration |  |  | B: Strattice |  |  |  |  |
| Rawlani et al. (2011)  Tissue expander breast reconstruction using prehydrated human acellular dermis. | Prospective observational | 121 reconstructions |  | B: Pre-hydrated acellular dermal matrix (ADM) | I | Expander-based extension of pec. maj. (2-stage) | Follow-up period after reconstruction was 44 ± 26.5 weeks | 9 (7.4%) soft-tissue infections, 8 (6.6%) partial mastectomy flap necroses, and 2 (1.7%) seromas.  11 (9.1%) explantations. Radiation pts trended to complications: 30.8% vs. 13.7%, P = 0.0749 |
| Bindingnavele et al. (2007)  Use of acellular cadaveric dermis and tissue expansion in postmastectomy breast reconstruction. | Retrospective observational | 41 pts (65 breasts) |  | B: Acellular cadaveric dermis | I | Expander-based extension of pec. maj. (2-stage) | Follow up: mean 10months (7-21m range)  “Good cosmetic outcome” | wound infection, expander removal, haematoma, and seroma were: 3.1% (two of 65), 1.5% (one of 65), 1.5% (one of 65), and 4.6% (three of 65), respectively |
| Zienowicz et al. (2007)  Implant-based breast reconstruction with allograft. | Prospective observational | 24 pts (30 breasts) | Skin- or nipple-sparing | B: AlloDerm | I | Single-stage/Direct to implant | Mean follow-up was 18 m (range, 15 to 24 m | No major skin flap necrosis. 6 minor skin-flap necrosis. No explantations. No rippling, symmastia, or sig bottoming-out. No capsular contracture ranking higher than Baker class I. |
| Liu et al. (2011)  Postoperative complications in prosthesis-based breast reconstruction using acellular dermal matrix. | Prospective observational | 470 reconstructions |  | B: Acellular dermal matrix vs None | I | Single + 2-stage (Implants or expanders) | Follow up: 5.5yrs | Trend to higher risk of major infections (therefore explantation) in ADM: 4.9% vs 2.5%, p = 0.172. Higher overall wound infection: 6.8 vs 2.5%, p = 0.031. BUT multivariate analysis: ADM use not sig RF for increased wound infection.  Higher surgical complication risk in ADM: 19.5vs12.3%, p<0.001). |
| Namnoum (2009)  Expander/implant reconstruction with AlloDerm: recent experience. | Prospective observational | 20 pts (29 breasts) | Nipple-sparing if possible | B: AlloDerm | I | Expander-based extension of pec. maj. (2-stage) | Follow up: mean 21m (3-32m) | 1 of 29 (3.4%) wound infection; 1 suture line necrosis + explantation. 1 seroma + minor op revision. |
| Losken (2009)  Early Results Using Sterilized Acellular Human Dermis (Neoform) in Post-Mastectomy Tissue Expander Breast Reconstruction. | Prospective observational | 22 pts (31 breasts) |  | B: NeoForm = Acellular human dermis | I | Expander-based extension of pec. maj. (2-stage) – in 16 pts | Follow-up: 10.2m (6-16m range) | 1 skin necrosis native to mastectomy + minor revision op. 1 explantation due to +ve deep margins. No cases of infection, foreign body reaction, rejection, seroma, or skin erythema. No encapsulation, extrusion, or infection of NeoFoam in 16 pts on re-exploration. |
| Spear et al. (2008)  Acellular dermis-assisted breast reconstruction. | Prospective observational | 43 pts (58 breasts) | Modified radical, simple, or nipple-sparing. | B: AlloDerm | I | Expander-based extension of pec. maj. (2-stage) | Stage 1 follow up: mean 25.9m (19.2–35.3 m range)  Stage 2 follow-up: 18.1m (6.7–31.6 m range) – 36 pts (83.7%) 50 breasts  Similar aesthetic outcome to non-surgery controls. | Stage 1 complication rate: 12%; 4 infections (+ 1 explantation), 2 partial mastectomy flap losses, and 1 seroma. Irradiation complication rate: with: 45.5% vs 4.3% without p = 0.002. Stage 2: 2.2%; 27.2% breast salvages due to irradiation; 1 Baker 3 capsular contracture + explantation. |
| Salzberg (2006)  Nonexpansive immediate breast reconstruction using human acellular tissue matrix graft (AlloDerm). | Prospective observational | 49 pts (76 breasts) | Simple total mastectomy, modified radical mastectomy, or subcutaneous mastectomy with nipple areolar preservation (skin-sparing). | B: AlloDerm | I | Single-stage | Follow-up: 18 (3–52 m range) | 1 pt full-thickness skin flap necrosis, w exposure of graft + surgical revision (closure). |
| Breuing et al. (2005)  Immediate bilateral breast reconstruction with implants and inferolateral AlloDerm slings. | Prospective observational | 10 pts (20 breasts) | Skin-sparing | B: AlloDerm | I | Single-stage | Follow-up: 6m-1yr range | 1 suture line ischemia + wound excision and closure. No contracture, infection, etc. But small sample size. |
| Gamboa-Bobadilla (2006)  Implant breast reconstruction using acellular dermal matrix. | Prospective observational | 11 pts (13 breasts) |  | B: AlloDerm | I: 62%; D: 38%. | Single-stage | Follow-up: 14 m (10–19 m range).  Aesthetic: 73% (8/11) pts excellent results, 18% (2/11) good, 9% (1/11) poor. | 9% complication rate: 1 pt seroma, cellulites + implant extrusion.  2 irradiated pts had no complications. |
| Breuing et al. (2007)  Inferolateral AlloDerm hammock for implant coverage in breast reconstruction. | Retrospective observational | 43 pts (67 breasts) – 10 pts included from 2005 paper |  | B: AlloDerm | I: 40 (30 single-stage, 10 2-stage); D (2-stage):4; Revision: 23 | Mixed Single-stage + 2-stage | Follow-up: 6m-3yrs | 4% (3/67): 2 infections (1 w explanation + revision – not counted as implant loss) + 1 implant loss due to implant extrusion (irradiated prior to reconstruction)  No haematoma, seroma, contracture) |
| Breuing et al. (2009)  Immediate breast tissue expander-implant reconstruction with inferolateral AlloDerm hammock and postoperative radiation: a preliminary report. | Retrospective observational | 5pts |  | B: AlloDerm | I | 1 single-stage; 4 2-stage | Follow-up: 2.5-5.5yrs post-reconstruction + 205yrs after post-reconstruction radiation | 1 small wound dehiscence + local excision and closure.  No infection, contracture or implant loss. Identical collagen architecture on histology from biopsy of implant capsule compared to non-radiated. |
| Preminger et al. (2008)  The influence of AlloDerm on expander dynamics and complications in the setting of immediate tissue expander/implant reconstruction: a matched-cohort study. | Matched retrospective cohort | 90 pts (45 matched pairs: expander size (±100 mL), history of prior irradiation, and indication for mastectomy (prophylactic/therapeutic). |  | B: AlloDerm cohort vs None cohort | I | Mixed single- + 2-stage | Follow-up not stated.  No increase in tissue expansion rate with AlloDerm. | No difference between AlloDerm + non-AlloDerm. Alloderm minor complications: 13.1% (6 of 45); cellulitis = 3, seroma = 3, hematoma = 1. |
| Salzberg et al. (2011)  An 8-year experience of direct-to-implant immediate breast reconstruction using human acellular dermal matrix (AlloDerm). | Retrospective observational | 260 pts (466 breasts) | Skin-sparing or nipple-sparing | B: AlloDerm | I | Single-stage | Follow-up: 28.9 ± 21.3 m (range, 0.3 to 97.7 m). | Overall 3.9%: implant loss 1.3%; skin breakdown/ necrosis, 1.1%; hematoma, 1.1%; human acellular dermal matrix exposure, 0.6%; capsular contracture, 0.4 %; and infection, 0.2%.  21 breasts (4.5%) had radiotherapy; 14.3% overall complication rate: 2 skin breakdown (+ 1 loss), 1 implant malposition w/o capsular contracture |
| Chun et al. (2010)  Implant-based breast reconstruction using acellular dermal matrix and the risk of postoperative complications. | Retrospective observational  No sig. differences btwn pt characteristics; except: Mean BMI + mastectomy specimen weight were higher in the ADM group | 283 pts (415 breasts) |  | B: 269 with + 146 w/o acellular dermal matrix | I | Mixed Single-stage + 2-stage | Follow-up not stated. | Seroma + infection rates were higher w ADM: infection: 14.1 vs 2.7% (p = 0.0003); seroma: 8.9 vs 2.1% (p = 0.0328). Multiple logistic regression analysis showed ADM + BMI were statistically sig. RFs for both. ADM increased the odds of seroma by 4.24 times (p = 0.018) + infection by 5.37 times (p = 0.006). |
| Topol et al. (2008)  Immediate single-stage breast reconstruction using implants and human acellular dermal tissue matrix with adjustment of the lower pole of the breast to reduce unwanted lift. | Prospective observational | 23 pts (35 breasts) | Skin-sparing | B: acellular dermal matrix | I | Single-stage | Follow-up: 9.5m (1-24m range) | 3 pts: 1 unilateral infection, implant exposure + revision; 1 bilateral infection + explantation; 1 unilateral implant exposure (no infection) + explantation |
| Nahabedian (2009)  AlloDerm performance in the setting of prosthetic breast surgery, infection, and irradiation. | Retrospective observational | 361 pts (476breasts)  With AlloDerm: 76 pts (100 breasts); W/o: 376 breasts |  | B: AlloDerm vs without | I: 60 pts; D: 3 for augmentation + 6 pts for revision reconstruction | 2-stage | Follow-up w AlloDerm: 17m (6-37m range) | Infection post-op: w/o: 5.85% (22/376); w AlloDerm = 5% (5/100);  Explantation: w AlloDerm: 2% (2/100); w/o: 5.32 (20/367).  Seroma: 5/100; Skin necrosis: 3/100; Incisional dehiscence: 4/100.  23/100 AlloDerm radiation: infection: 8.7% (2/23); seroma: 3/23; skin necrosis: 0/23; incisional dehiscence: 3/23. |
| Nguyen et al. (2010)  Infectious Complications Leading to Explantation in Implant-Based Breast Reconstruction With AlloDerm. | Retrospective cohort | 321 breasts: 75 with AlloDerm; 246 w/o |  | B: AlloDerm vs w/o | I: w AlloDerm = 72; w/o = 192.  D: w AlloDerm = 4; w/o = 54. | 2-stage | Follow-up: not stated | Readmission for IV antibiotics: (AlloDerm) 2.8% vs (w/o) 5.3%; p = 0.291 (no sig diff).  Explantation due to infection, seroma, extrusion: 8.0% (n = 6) vs 1.6% (n = 4), p=0.013 (sig higher in AlloDerm). |
| Lanier et al. (2010)  The effect of acellular dermal matrix use on complication rates in tissue expander/implant breast reconstruction. | Retrospective cohort | 127 breasts |  | B: acellular dermal matrix vs none  ADM = 75; none: 52. | I | 2-stage | Follow-up: not stated | ADM higher rate of infection: 28.9% vs. 12.0% (P = 0.022), reoperation: 25.0% vs. 8.0%, (P = 0.011), expander explantation: 19.2% vs. 5.3% (P = 0.020), and overall complications: 46.2% vs. 22.7% (P = 0.007). |
| Basu et al. (2010)  Acellular cadaveric dermis decreases the inflammatory response in capsule formation in reconstructive breast surgery. | Prospective observational | 20 pts |  | B: AlloDerm |  | 2-stage | Intraoperative biopsy during implant exchange for histopathologic analysis: AlloDerm statistically sig lower inflammatory changes (observed granulation tissue formation, vessel proliferation, chronic inflammatory changes, capsulefibrosis, fibroblast cellularity, and foreign body giant cell inflammatory reaction) that initiate capsule formation than controls (p<0.001). |  |
| Wong et al. (2008)  Histologic analysis of angiogenesis and lymphangiogenesis in acellular human dermis. | Time course + mechanism of vascularisation + tissue incorporation of AoolDerm in a rat. |  |  | B: AlloDerm |  | Flap: like superficial inferior epigastric pedicle technique | The host response to AlloDerm parallels normal wound healing. Host cell infiltrate increases steadily over a 14-day period. By 7 days after implantation, a large number of CD31 endothelial cells have infiltrated the matrix and early vessels are abundantly present. These vessels continue to mature by day 14. Finally, the authors show that AlloDerm composite flaps also support infiltration and development of a lymphatic network. |  |
| Spear et al. (2011)  Acellular dermal matrix for the treatment and prevention of implant-associated breast deformities. | Retrospective observational | 52 pts (77 breasts) |  | B: acellular dermal matrix | 1⁰, 2⁰ + breast reconstruction using ADM + implants (single –stage) | Indications included prevention of implant bottoming-out (n = 6), treatment of malposition (n = 32), rippling (n = 20), capsular contracture (n = 16), and skin flap deficiency (n = 16). | Follow-up: 8.6m mean (0.4-30.3m range).  74 breasts (96.1%) managed successfully with ADM. | 3 failures: 1 breast with bottoming-out following treatment of capsular contracture, 1 breast with major infection requiring device explantation, and 1 breast with recurrent rippling.  9.1% total complication rate: 3 mild infections, 1 major infection + explantation, 1 hematoma, and 1 seroma. |
| Komorowska-Timek et al. (2009)  The effect of AlloDerm envelopes on periprosthetic capsule for mation with and without radiation. | Two 5-ml implants were placed submuscularly in the backs of 41 rats. The right implant was wrapped with AlloDerm and the left remained bare. | 20 irridiated to each implant, 21 no radiation |  | B: AlloDerm |  |  | Radiation increased inflammation of bare capsules at 12 weeks, but it was significantly reduced in irradiated AlloDerm capsules. The majority of irradiated bare capsules developed pseudoepithelium, whereas AlloDerm protected capsules from this transformation.  AlloDerm decreases radiation-related inflammation and delays or diminishes pseudoepithelium formation and thus may slow progression of capsular formation, fibrosis, and contraction. |  |
| Pozner et al (2013)  Use of porcine acellular dermal matrix in revisionary cosmetic breast augmentation. | Retrospective chart review | 93 consecutive patients (179 breasts |  | B= Strattice | cosmetic breast augmentation |  | Average follow-up was 12 months (range, 1-39 months). | There were 2 major complications (1.6% of breasts): an infection in 1 breast that required implant explantation approximately 2 weeks postoperatively and an extrusion that required PADM removal. Two additional patients had high-riding implants resulting from folded PADM that required revision; both cases were corrected by excising the folded PADM segment. Seven other patients required office procedures to correct minor imperfections. Two CC recurrences were suspected (1 patient) in the 76 breasts that underwent capsulectomy and PADM placement. |
| Bengtson (2013)  Discussion: Use of Dermal Matrix to Prevent Capsular Contracture in Aesthetic Breast Surgery | Discussion  (must read) |  |  |  |  |  |  | Capsular contracture |
| Kolt et al. (2013)  The Use of Human Acellular Dermal Matrix in the First Stage of Implant-Based Breast Reconstruction Simplifies the Exchange Procedure | Retrospective review | 57 pts (92 breasts) w ADM + 15 pts (17 breasts) w/o |  | B: Acellular dermal matrix | I | 2-stage | The increased pocket control afforded by ADM will simplify the second stage of the operation by decreasing the need for and extent of capsular modifications. Capsular procedures were graded as follows: 0, no revision; 1, capsulotomy; 2, capsulectomy; and 3, capsulorrhaphy.  Frequency of capsule revision grades in the human ADM versus non–ADM groups, respectively, were: 0, 40 (43%) versus four (24%), p = 0.04; 1, 23 (25%) versus eight (47%), p = 0.06; 2, 24 (26%) versus four (24%), p = 0.62; and 3, five (5%) versus one (5 %), p = 0.81. The odds ratio of having a lower capsular score given a unit increase in non–acellular dermal matrix use was 3.1 (p = 0.2). |  |
| Potter et al. (2013)  Early complications and implant loss in implant-based breast reconstruction with and without acellular dermal matrix (Protexa®): A comparative study. | Retrospective review | 31 pts (46 breasts) | Skin or nipple-sparing | B: ADM (Protexa) | I | Mix:  Single-stage w ADM: 25 breasts (80.6%)  2-stage: ADM: 6 breasts (19.4%); none: 15 breasts (32.6%) |  | 12 (26.7%) early complications; 4 (8.7%) haematomas, 4 (8.7%) wound dehiscences, 2 (4.3%) seromas and 2 (4.3%) wound infections requiring antibiotics but there were no significant differences in the rate of complications between the procedure groups (n = 4, 26.7% standard group vs. n = 8, 25.8% ADM group, p = 0.950, chi-squared test). The odds ratio for early complications in the presence of ADM was 0.96 (95% confidence interval 0.23–3.94). Major complications included 2 (8.7%) haematomas requiring evacuation; both in patients receiving standard expander-implant reconstruction. odds ratio for major complications in the ADM group was 0.22, but this was not statistically significant (95% confidence interval 0.02–2.81).  Six implants (13.0%) were removed due to infection within three months of surgery; two (13.3%) in the standard expander group and four (12.9%) in patients receiving ADM-assisted procedures (p = 0.968, chi-squared test). The odds ratio for implant loss in the ADM group compared with patients receiving the standard procedure was 0.96 (95% confidence interval 0.15–6.08). All implant losses occurred in women receiving bilateral reconstruction and all implant losses in the ADM group were associated with pre-reconstruction radiotherapy to the breast. Univariable logistic regression provided strong evidence that previous radiotherapy was associated with a significantly increased risk of implant loss (odds ratio 14.0, 95% confidence interval 2.01–97.3, p = 0.008). |
| Lee et al. (2013)  A Comparative Study of CG CryoDerm and AlloDerm in Direct-to-Implant Immediate Breast Reconstruction. | Retrospective review | 50 pts/breasts | Skin-sparing or nipple-areolar sparing | B: 31 AlloDerm vs 19 CryoDerm | I | Single stage | Follow-up Mean 17m (AlloDerm) + 14m (CryoDerm) | no significant differences in the overall incidence of complications (seroma, infection, skin flap necrosis, capsular contracture, and implant loss) between the two groups.  In the AlloDerm group, the overall incidence of complications was 22.6% (7/31); these included five cases (16.1%) of seroma, four cases (12.9%) of infection (defined as a condition where the use of intravenous antibiotics was needed), one case (3.2%) of skin flap necrosis, and one case (3.2%) of capsular contracture (Baker grade III or IV). One case (3.2%) of implant loss was secondary to skin flap necrosis followed by uncontrolled infection. In the CG CryoDerm group, the overall incidence of complications was 26.3% (5/19); these included one case (5.3%) of seroma, four cases (21.1%) of infection, and one case (5.3%) of capsular contracture (Baker grade III/IV). One case (5.3%) of implant loss secondary to implant rupture at 1 year after surgery was reconstructed with autologous tissue. |
| Cayci et al. (2013)  Impact and outcome of human acellular dermal matrix size for immediate and two-stage breast reconstruction. | Retrospective observational  Pts divided by matrix size | 52 pts (88 breasts) |  | B: ADM | I | 2-stage | Mean follow-up was 49.5 months (range, 42 to 63 months). | 7pts: overall incidence of 13.2%. No sig diff btwn 2 groups. 1 seroma, 1 haematoma, 2 infections, 2 skin flap necrosis, 1 tissue expander rupture. |
| Wu et al. (2013)  Human acellular dermal matrix (AlloDerm®) dimensional changes and stretching in tissue expander/implant breast reconstruction. | Prospective case series  Compared to similar cohort w/o human ADM | 31 pts  45 pts |  | B/ADM | I | 2-stage | Follow up: 96 days in human ADM group | 3 pts in HADM group (10%): 1 seroma, 1 UTI, 1 red breast syndrome (delayed erythema on skin overlying implant.  6 pts in control (13%): 2 cellulitis, 2 expander replacement (1 leak + 1 exposure), 1 delayed wound healing, 1 skin necrosis. |
| Michelotti et al. (2013)  Analysis of clinically significant seroma formation in breast reconstruction using acellular dermal grafts. | Retrospective review | 73 pts (284 breasts)  220 breasts w ADM, 64 none. | Nipple-sparing, wise pattern, or skin-sparing | B: AlloDerm (AL), DermaMatrix (DM), and FlexHD (FHD) or None. | Mixed | 2-stage |  | Overall: 17 (7.7%) seromas. AL (n = 2, 4.0%), DM (n = 6, 5.4%), FHD (n = 9, 14.75%), and no ADM (n = 1, 1.5%). Sig difference btwn product types: p=0.016. Multivariate analysis identified a strong trend toward FHD as an independent predictor of seroma formation (P = 0.061).  The percentage of patients receiving either preoperative or postoperative radiation was AL 24%, DM 12%, FHD 25%, and no ADM 6% |
| Weichman et al. (2013)  Sterile "ready-to-use" AlloDerm decreases postoperative infectious complications in patients undergoing immediate implant-based breast reconstruction with acellular dermal matrix. | Prospective observational | 546 breasts |  | B/ADM  64.3 percent (n = 351) with no acellular dermal matrix, 16.5 percent (n = 90) with aseptic matrix, and 19.2 percent (n = 105) with ready-to-use matrix. | I | Single-stage |  | Ready-to-use vs aseptic: decrease in overall infection (8.5 percent versus 20.0 percent; p = 0.0088), major infection (4.7 percent versus 12.2 percent; p = 0.069), and need for explantation (1.9 percent versus 6.6 percent; p = 0.1470). |
| Ohkuma et al. (2013)  Initial experience with the use of foetal/neonatal bovine acellular dermal collagen matrix (SurgiMend™) for tissue-expander breast reconstruction. | Retrospective review | 64 pts (95 breasts) |  | B: SurgiMend | I | 2-stage | Mean follow-up time was 16.9 ± 8.7 months | haematoma 3.2%, seroma 7.5% and 3 infections (+ 2 re-operation due to infection 2.1%). |
| Reish et al. (2013)  Infection following implant-based reconstruction in 1952 consecutive breast reconstructions: salvage rates and predictors of success. | Retrospective analysis | 1241 pts (1952 breasts) |  | B/ADM | I | 2-stage |  | 94 pts/99 breasts/5.1% had erythema = poss infection. Had a higher incidence of smoking (p = 0.007), chemotherapy (p = 0.007), irradiation (p = 0.001), and mastectomy skin necrosis (p < 0.0001). 74 pts (74.7 %) attempted salvage op (n = 18) or explantation (n = 56). There was no difference in acellular dermal matrix use between these two groups. Salvage with intravenous antibiotics and implant exchange was successful in 37.3 % of patients. |
| Liu et al. (2013)  Comparison of Outcomes Using AlloDerm Versus FlexHD for Implant-Based Breast Reconstruction. | Retrospective | 382 pts (547 breasts) |  | B: 175 used AlloDerm and 113 used FlexHD. Vs 177 none | I: 81.5% + D | Mix – mostly 2-stage | Follow-up: 6.4 m mean | Overall Return to OR 47 breasts (8.6%); Surgical site infection 53 (9.7); Seroma 33 (6.0); Hematoma 6 (1.1); Delayed healing/flap necrosis 76 (13.9); Implant loss 38 (6.9); Implant malposition 9 (1.6).  Implant deflation 5 (0.9) |
| Butterfield (2013)  440 Consecutive immediate, implant-based, single-surgeon breast reconstructions in 281 patients: a comparison of early outcomes and costs between SurgiMend fetal bovine and AlloDerm human cadaveric acellular dermal matrices. | Retrospective analysis | 281 pts (440 breasts) |  | B: SurgiMend [222 pts, 351 breasts (79.0 percent)] or AlloDerm [59 pts, 89 breasts (21.0 percent)] | I | Mixed – mostly 2-stage | Follow-up: min 3m | SurgiMend vs AlloDerm: Haematoma: 4 breasts (1.1%) vs 0; Seroma:30 (8.6.) vs 14 (15.7); Necrosis: 39 (11.1) vs 3 (3.4); Infection: 17 (4.8) vs 6 (6.7); Expander/implant loss: 29 (8.3) vs 10 (11.2). No sig diff btwn major complications. SurgiMend cheaper. |
| Buseman et al. (2013)  Comparison of sterile versus nonsterile acellular dermal matrices for breast reconstruction. | Retrospective analysis | 58 pts | Modified radical, skin-sparing, and nipple-sparing | B/ADM: 9 sterile, 25 aseptic (original), 24 no ADM. | I | Mixed |  | Most frequent complication was seroma: in 6/9 patients with sterile ADM as compared to 2/25 with the aseptic ADM. This was statistically significant (P = 0.003).  Etiology of this increased incidence remains unknown, but it correlates with the introduction of the sterile form of ADM at our institution. A different preparation or sterilization process, or some other variable as yet unknown, may be responsible. |
| Pestana et al. (2013)  Factors affecting complications in radiated breast reconstruction. | Retrospective analysis | 154 pts (157 breasts) |  | B: 24% w ADM (AlloDerm) | I: 109; D:48. |  | Mean follow-up: 6yrs | When ADMs were used in conjunction with radiotherapy, a significant increase in the incidence of a major complication was identified. 65% pts w ADM vs 45% pts w/o ADM use. ADM 2.3 times more likely to need reoperation (P = 0.03) |
| Brzezienski et al. (2013)  Classification and management of seromas in immediate breast reconstruction using the tissue expander and acellular dermal matrix technique. | Retrospective analysis | 67 pts (100 breasts) |  | B: ADM | I | 2-stage |  | 31 clin sig. Seromas. 3/100 (3%) TE explantation – only 1 due to seroma. |
| Pannucci et al. (2013)  The impact of acellular dermal matrix on tissue expander/implant loss in breast reconstruction: an analysis of the tracking outcomes and operations in plastic surgery database. | Retrospective analysis | 14,249 pts – complete data on 13,957 pts |  | B/ADM: 3450 (24%) | Mix | Mix | Follow-up: 30days | Overall expander/implant loss: 292/13,957 (2.05%); ADM: absolute risk increase of 0.7% for expander/implant loss (2.58 % vs 1.88 %, p = 0.012). |
| Gaster et al. (2013)  Histologic Analysis of Fetal Bovine Derived Acellular Dermal Matrix in Tissue Expander Breast Reconstruction. | Prospective observational | 12 pts (17 breasts) |  | B: SurgiMend | I | 2-stage | Biopsy taken at exchange – time to exchange/implantation time: 7.8m mean (2-23m range).  Follow-up: 2yrs | 1 infection + TE explantation. Contracture, inflammatory changes, edema, and polymorphonuclear leukocyte infiltration were rare in the ADM. An acellular capsule was seen in many cases, at the interface of SurgiMend with the tissue expander. |
| Avashia et al. (2013)  Postoperative antibiotic prophylaxis for implant-based breast reconstruction with acellular dermal matrix. | Retrospective analysis | 84 pts (119 breasts) |  | B: AlloDerm | Mix | 2-stage | Follow-up: 3yrs | Rates of postop infection requiring TE removal were 7.9% + 3.2% for both experimental cohorts (administration of antibiotics >=48 hours) vs 31.6 % in control group (p = 0.004). |
| Seth et al. (2013)  A comparative analysis of cryopreserved versus prehydrated human acellular dermal matrices in tissue expander breast reconstruction. | Retrospective  analysis | 255 pts (369 breasts) |  | B: 136 cryopreserved vs 233 prehydrated HADM | I | 2-stage | Follow-up: 5yrs | Total cryopreserved vs prehydrated HADMs were 19.1% and 19.3% (P = 1.0). Flap necrosis (8.1% vs 9.0%, P = 0.849), infection requiring intravenous antibiotics (10.3% vs 5.2%, P = 0.09), hematoma (2.9% vs 1.3%, P = 0.431), seroma (2.2% vs 1.0%, P = 1.0), expander exposure/dehiscence (5.9% vs 6.4%, P = 1.0), and number of autologous reconstruction due to complication (4.4% vs 6.4%, P = 0.491). On regression analysis, HADM type was not an independent risk factor for any complication subtype. |
| Davila et al. (2013)  Human Acellular Dermis versus Submuscular Tissue Expander Breast Reconstruction: A Multivariate Analysis of Short-Term Complications. | Retrospective analysis | 9,159 pts |  | B/ADM in 1,717 + none in 7,442 (submuscular placement) | I | 2-stage |  | Reconstruction-related = similar in both (ADM vs None): 4.7% vs. 4.3%, P=0.39.  Infection: 3.8 vs 3.3; Wound disruption: 0.3 vs 0.5; 1 vs 0.8. No significant p values. |
| Semprini et al. (2013)  The bovine pericardial patch in breast reconstruction: a case report. | Case report | 1 pt | Nipple-sparing | B: Tutopatch® | I | 1-stage | Follow-up: 12m | No complications |
| Salzberg (2012)  Focus on technique: one-stage implant-based breast reconstruction. | Retrospective | 439 pts (90 breasts) |  | B/ADM | I | 1-stage | Mean follow-up, 3.2 years; range, 0.1 to 10.1 years. | Implant loss (1.6 %), skin necrosis requiring reoperation (1.3 %), infection (1.1 %), hematoma (0.6 %), seroma (0.5 %), and capsular contracture (0.5 %) |
| McCarthy et al. (2012)  The use of acellular dermal matrices in two-stage expander/implant reconstruction: a multicenter, blinded, randomized controlled trial. | RCT | 70 pts |  | B/ADM vs Submuscular | I | 2-stage | No differences in immediate postoperative pain (p = 0.19) or pain during the expansion phase (p = 0.65) between treatment arms. There was no difference in postoperative narcotic use (p = 0.38). | No diff btwn 2 groups ADM: 6 (1.7%) vs Submuscular: 5 (1.5%) (p=1.00). Haematoma: 1 vs 1; Seroma: 1 vs 3; Infection 3 vs 1; Premature removal of device: 1 vs 0 pts. |
